# Contrasting glucosinolate profiles in rapeseed genotypes shape the rhizosphere-insect continuum and microbial detoxification potential in a root herbivore

**DOI:** 10.1128/msystems.01269-25

**Published:** 2025-11-17

**Authors:** J.M. Carpentier, S. A. P Derocles, S. Chéreau, B. Marquer, J. Linglin, L. Lebreton, F. Legeai, N. Vannier, A.M. Cortesero, C. Mougel

**Affiliations:** 1 IGEPP, INRAE, Institut Agro, Univ Rennes, Le Rheu, France; 2 Univ Rennes, CNRS, ECOBIO [(Ecosystèmes, Biodiversité, Evolution)] - UMR 6553, Rennes, France; University of Pretoria, Hatfield, South Africa

**Keywords:** plant-insect-microbiome interactions, *Delia radicum*, *Brassica napus*, *Pseudomonas brassicacearum*, horizontal transmission, glucosinolates, isothiocyanates

## Abstract

Plant secondary metabolites are key mediators of plant–insect–microbiome interactions, yet their role in structuring functionally relevant insect-associated microbial communities remains poorly understood. Here, we combined a factorial experiment using *Brassica napus* genotypes differing in glucosinolate (GLS) content with distinct succession to investigate the eco-evolutionary dynamics of the microbiota of the root herbivore *Delia radicum*. Amplicon sequencing and microbial culturing revealed that both rhizospheric and gut microbial communities are shaped by plant genotype and soil legacy, with a subset of bacterial taxa shared across compartments. Notably, *Pseudomonas brassicacearum*, harboring the isothiocyanates (ITC) detoxifying gene *saxA*, was consistently recovered from both plant and insect habitats. Functional assays confirmed its capacity to degrade 2-phenylethyl isothiocyanate (PEITC), a major toxic GLS hydrolysis product. Other gut-derived microbial isolates exhibited heterogeneous responses to PEITC, ranging from growth inhibition, promotion, or growth recovery after a prolonged lag phase. Despite the toxicity of ITC, insect fitness proxies were enhanced on GLS +plants, suggesting microbiota-mediated adaptation to host chemical defenses. Our findings reveal a plant genotype-specific filtering of environmentally acquired microbes and highlight the role of detoxifying symbionts in *Delia radicum* performance.

**IMPORTANCE:**

Understanding how herbivorous insects adapt to plant chemical defenses is important in the context of new agricultural practices. This study highlights that the host plant genotype shapes not only rhizospheric and gut microbial communities but also promotes the acquisition of symbiotic bacteria capable of detoxifying harmful isothiocyanates. These findings reveal a functional microbial pathway for insect adaptation to plant defenses, with potential implications for pest management strategies. By uncovering the role of plant-associated microbiota, the acquisition of beneficial microbes, and their functional contributions to host fitness, this work provides a foundation for innovative agroecological approaches that leverage plant–microbe–insect interactions.

## INTRODUCTION

Plants have evolved diverse mechanisms to protect themselves from herbivory. These defensive strategies include both morphological structures and the production of toxic specialized metabolites that can act as repellents, digestive inhibitors, or direct toxins for herbivorous insects (1). In response, insects have developed a wide range of adaptations including physiological detoxification (2), behavioral avoidance (3), sequestration (4), excretion (5), and enzymatic detoxification (6).

While endogenous detoxification is well described across many insect taxa (7–9), increasing evidence highlights the role of microbial symbionts in supporting these detoxification processes. For instance, bacteria isolated from the mountain pine beetles (*Dendroctonus ponderosae*) can degrade terpenes and improve host fecundity (10). Similarly, in the gypsy moth (*Lymantria dispar*), bacterial strains isolated from aspen leaves enhanced larval growth only in the presence of phenolic glycosides, suggesting a detoxification function (11). In the olive fly (*Bactrocera oleae*), symbionts such as *Candidatus* species and *Erwinia aphidicola* have been shown to express genes involved in oleuropein degradation (12).

Microbial symbionts can be transmitted vertically (from parent to offspring) or horizontally (acquired from the environment) (13, 14). While vertical transmission of nutritive and defensive symbionts is well studied (e.g. [15, 16]), horizontal acquisition of plant toxin-degrading symbionts is less documented. Environmental microbiota, particularly in the rhizosphere, may serve as a reservoir for microorganisms able to thrive in toxin-rich plant environments (11, 17).

In this context, we focused on the cabbage root fly, *Delia radicum* (Diptera: Anthomyiidae), a phytophagous insect specializing on Brassicaceae, and investigated its potential to adapt to plant chemical defense via its microbiota. Brassicaceous plants deploy a chemical defense system based on glucosinolates (GLS) and the myrosinase enzyme. Upon tissue damage, myrosinase hydrolyzes GLS into an unstable aglucone form that spontaneously transforms into isothiocyanates (ITC, 18). ITC exhibit strong insecticidal effects, including growth inhibition (19), delayed development (20), metabolic disruption (21), and increased mortality (22). Insects may mitigate these effects through both endogenous pathways such as cytochrome P450s (23, 24) and microbiome-mediated detoxification (25).

*Delia radicum* females lay eggs near the stem of Brassicaceous plants, and the larvae feed on the roots while developing in the rhizosphere (26). This species is holometabolous; therefore, its microbial communities are different across multiple life stages (27). Previous studies have characterized its bacterial communities, which are typically dominated by *Wolbachia*, a vertically transmitted endosymbiont (27–30). Other bacterial taxa, including *Serratia* sp., *Pectobacterium* sp., *Acinetobacter* sp., and *Providencia* sp., have also been isolated from the larval gut and shown to be able to degrade ITC *in vitro* (31). Notably, a *Pectobacterium* strain harboring the *saxA* gene, an ITC-detoxifying gene, was able to hydrolyze 2-phenethyl isothiocyanate (PEITC) (31). While these findings suggest a role for toxin-degrading symbionts in insect adaptation to chemical defenses, the acquisition routes and fitness impact of these microbial symbionts remain unclear.

This study aimed to identify the microbial taxa shared between *D. radicum* and its environment (soil and plant roots) and to assess the contribution of environmental microorganisms to ITC detoxification. We further sought to determine whether acquiring these microbes confers a fitness advantage to *D. radicum* when feeding on high-GLS plants.

To address these questions, we designed a laboratory experiment exposing *D. radicum* to different soil types and rapeseed (*Brassica napus*) genotypes. Two *B. napus* genotypes were selected from a panel of 300, based on their contrasting GLS content (32): the Romeo genotype (high GLS, referred to as GLS+) and the Bronowski genotype (low GLS, referred to as GLS−).

Plants apply a selection pressure to rhizosphere microbial communities during their growth by selecting taxa adapted to the plant-produced compounds, sculpting a soil legacy (33, 34). Rapeseed could thus potentially favor soil microbiota adapted to ITCs (35, 36). Consequently, the two rapeseed genotypes were grown on three soils with different microbial legacies, originating from distinct successions of plants producing different levels of GLS.

The specific objectives of this study were as follows: (i) to assess the impact of soil legacy and rapeseed genotype on microbiota diversity by characterizing bacterial and fungal communities in the rhizosphere, roots, *D. radicum* larvae, and adults using metabarcoding; (ii) to detect potential horizontal transmission, by comparing microbial communities across the rhizosphere, roots, and *D. radicum* larvae; (iii) to assess the tolerance of larval gut-associated microorganisms to PEITC, by comparing growth kinetics across gut isolates; (iv) to identify microorganisms involved in ITC detoxification, by isolating gut bacteria from larvae, screening for the presence of the *saxA* gene by PCR, sequencing and analyzing sequences of *saxA* genes, and testing their ITC-degrading capacities *in vitro*; (v) to evaluate the effect of microbial diversity on insect performance, by measuring larval and adult fitness traits (morphometric and survival) as indicators of adaptation to a GLS environment.

## RESULTS

### Plant metabolites

To assess whether plant root metabolites differed across the experimental modalities (GLS−/Wheat, GLS−/GLS−, GLS+/Wheat, and GLS+/GLS+), we quantified glucosinolates (GLS), digestible carbohydrate, and soluble protein in roots.

Mean GLS concentrations varied significantly among modalities (Kruskal-Wallis test, χ² = 97.4, df = 3, *P* < 0.001) with both GLS+/GLS + and GLS+/Wheat showing higher GLS concentrations compared to GLS−/GLS− and GLS−/Wheat (Fig. S1). This confirms that our experimental design effectively contrasted two plant genotypes with different GLS contents.

Digestible carbohydrate concentrations also differed across modalities (Kruskal-Wallis test, χ² = 23.56, df = 3, *P* < 0.001) with the GLS+/GLS +modality exhibiting significantly higher values than all others (Fig. S2A).

In contrast, soluble protein concentrations did not vary significantly among modalities (Kruskal-Wallis test, χ² = 4.57, df = 3, *P* = 0.206, Fig. S2B). These results indicate that the experimental modalities generated distinct chemical environments within the roots, particularly in terms of GLS and carbohydrate availability, while soluble protein concentrations remained unaffected.

### Effect of soil legacy and rapeseed genotype on rhizosphere bacterial and fungal communities

To investigate how previous plant cultivation (soil legacy) and rapeseed genotype influence the composition and diversity of rhizosphere microbial communities, we characterized both bacterial and fungal communities in soil and root compartments. We analyzed community richness, diversity, and taxonomic composition and used multivariate approaches to assess differences in overall community structure among soil legacies and plant genotypes.

### Alpha diversity

To compare microbial diversity between compartments, we analyzed both bacterial and fungal communities in rhizospheric soil and roots using the number of observed ASVs and Shannon diversity index. Bacterial diversity strongly differed between compartments. Both the number of observed ASVs and Shannon Index were significantly higher in rhizospheric soil compared to roots (Kruskal-Wallis test, χ² = 67.39, *P* < 0.001), confirming that rhizospheric soil harbored a richer and more even bacterial community than the root compartment.

**Fig 1:**
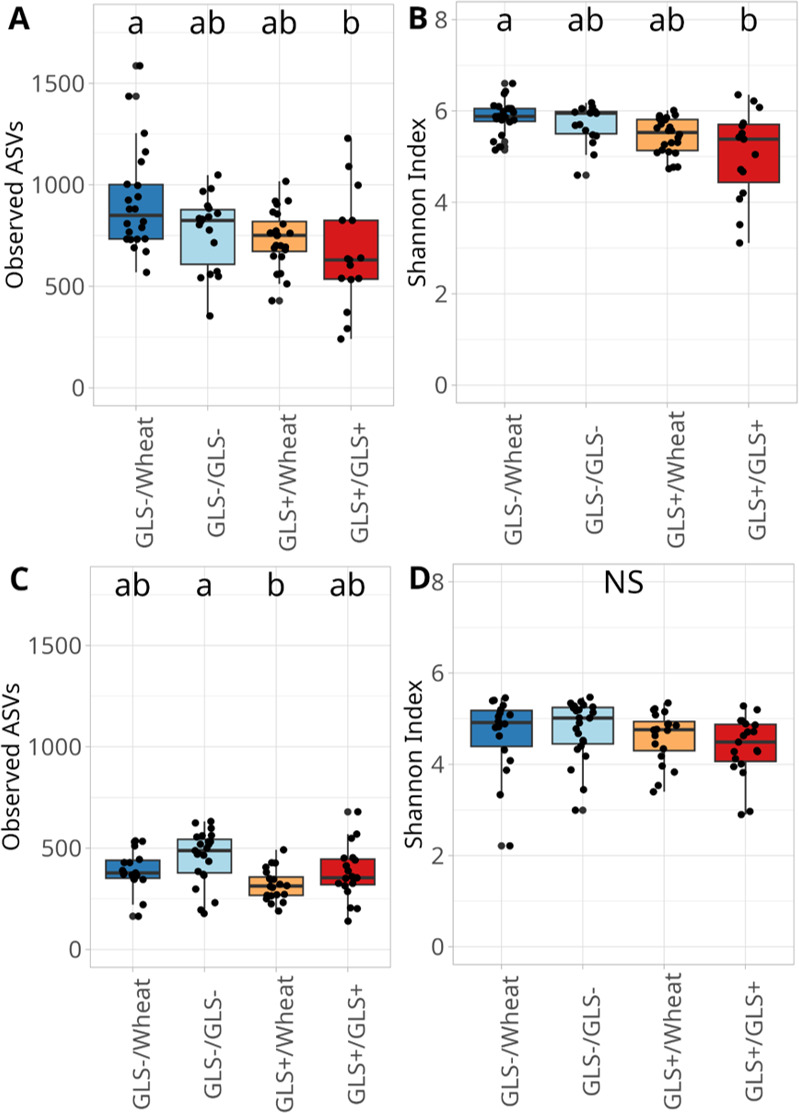
Observed ASVs and Shannon index of bacterial communities in soil (**A and B**) and roots (**C and D**). The four modalities were derived from combining two rapeseed genotypes (GLS+ and GLS−) with three soil legacy conditions (GLS−, GLS+, and Wheat), resulting in the following four modalities: GLS−/Wheat, GLS−/GLS−, GLS+/Wheat, and GLS+/GLS+. Data were analyzed using the Kruskal-Wallis test. Different letters indicate a significant difference where *P* < 0.05.

**Fig 2:**
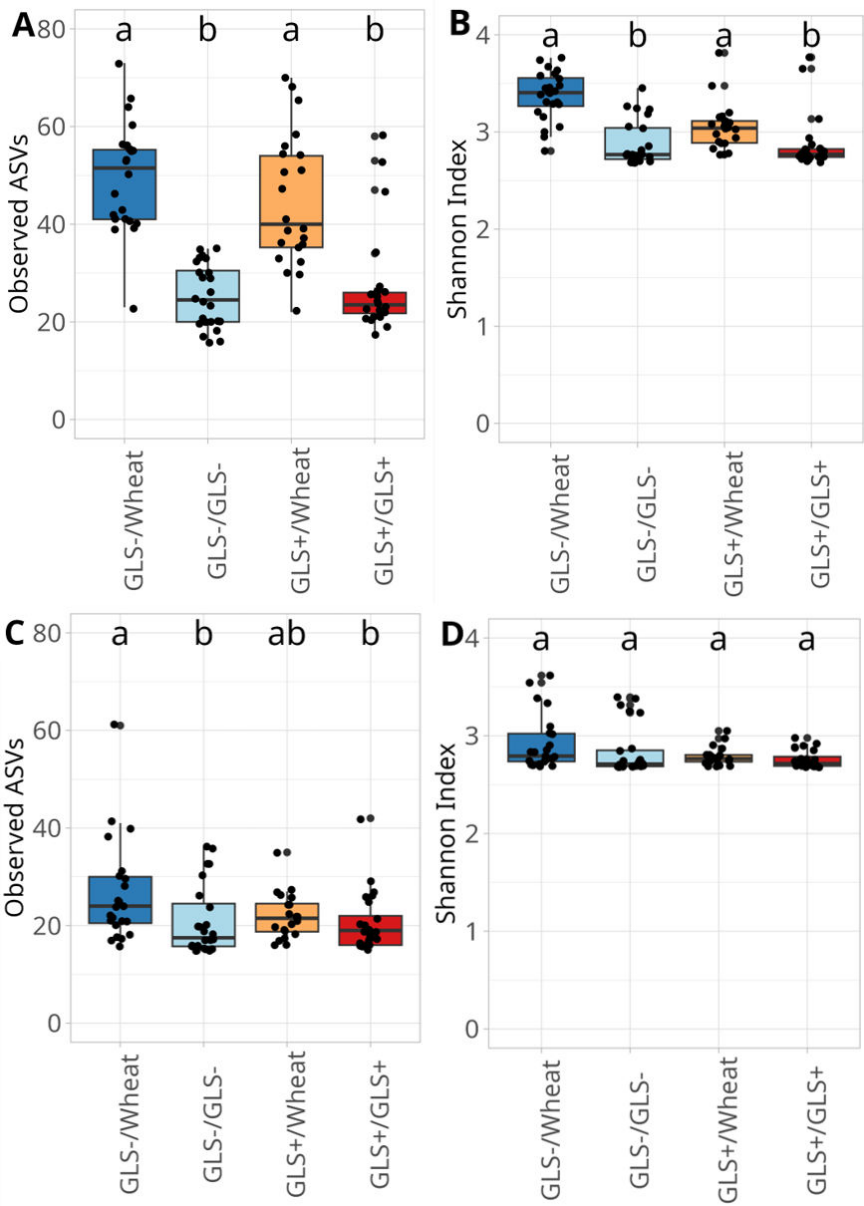
Observed ASVs and Shannon index of fungal communities in soil (**A and B**) and roots (**C and D**). The four modalities were derived from combining two rapeseed genotypes (GLS+ and GLS−) with three soil legacy conditions (GLS−, GLS+, and Wheat), resulting in the following four modalities: GLS−/Wheat, GLS−/GLS−, GLS+/Wheat, and GLS+/GLS+. Data were analyzed using the Kruskal-Wallis test. Different letters indicate a significant difference, where *P* < 0.05.

Within the soil, bacterial richness significantly varied among plant modalities (Kruskal-Wallis test, χ² = 10.68, df = 3, *P* = 0.014) with GLS−/Wheat showing the highest richness compared to GLS+/GLS+ (Fig. 1A). The same results were observed for the Shannon index (Kruskal-Wallis test, χ² = 15.06, df = 3, *P* = 0.0017, Fig. 1B), indicating that soil microbial communities in the GLS−/Wheat modality were richer. In roots, bacterial richness also differed across modalities (Kruskal-Wallis test, χ² = 15.79, df = 3, *P* = 0.0125), with higher values in GLS−/Wheat than GLS+/Wheat (Fig. 1C). However, the Shannon Index in root-associated communities was not significantly affected by modalities (Kruskal-Wallis test, χ² = 6.006, df = 3, *P* = 0.11, Fig. 1D), suggesting that bacterial diversity did not vary across conditions. Fungal communities exhibited similar trends. Soil fungal richness significantly differed among modalities (Kruskal-Wallis test, χ² = 51.39, df = 3, *P* < 0.01) with GLS−/Wheat and GLS+/Wheat hosting richer fungal communities than both GLS−/GLS− and GLS+/GLS+ (Fig. 2A). Shannon diversity followed the same pattern (Kruskal-Wallis test, χ² =41.2, df = 3, *P* < 0.001, Fig. 2B). In roots, fungal community richness was also affected by modalities (Kruskal-Wallis test, χ² =10.9, df = 3, *P* = 0.012), with significantly higher values in GLS−/Wheat compared to GLS−/GLS− and GLS+/GLS+ (Fig. 2C). Although a significant effect was detected for the Shannon index (Kruskal-Wallis test, χ² = 8.31, df = 3, *P* = 0.034, Fig. 2D), pairwise post-hoc comparisons did not reveal differences among modalities.

Together, these results demonstrate that compartment (soil and root), plant genotype, and the soil legacy strongly shape microbial diversity. The wheat-associated modalities, particularly GLS−/Wheat, had higher bacterial and fungal richness in both soil and roots, whereas GLS +conditions were associated with reduced microbial diversity.

### Composition of microbial communities

We characterized the taxonomic composition of bacterial and fungal communities in both rhizospheric soil and roots to identify the dominant microbial phyla.

Soil bacterial communities were primarily composed of Proteobacteria, Bacteroidetes, Actinobacteria, Firmicutes, Verrucomicrobia, and Parcubacteria (Fig. S3A). In contrast, root-associated bacterial communities displayed a less complex composition, dominated by Proteobacteria, Bacteroidetes, Firmicutes, and Actinobacteria (Fig. S3B). This reduction in taxonomic diversity from soil to root indicates a strong filtering effect exerted by the plant compartment, whereby only a subset of soil bacterial taxa is able to colonize the root environment.

Fungal communities were dominated by Olpidiomycota, represented exclusively by *Olpidium brassicae*, and Ascomycota in both soil (Fig. S4A) and roots (Fig. S4B). The prevalence of *O. brassicae* across compartments suggests specialization toward *Brassica napus*, whereas Ascomycota contributed a more diverse but less dominant component.

These results highlight that while bacterial communities showed a compartment-driven shift in dominant phyla, fungal communities were characterized by the consistent dominance of *O. brassicae* across rhizospheric soil and roots, suggesting a strong selective pressure on fungal community structure.

### Beta diversity

To assess how host plant modalities influenced microbial community structure, we performed distance-based redundancy analyses (dbRDA) and permutational multivariate analysis of variance (PERMANOVA) on Bray-Curtis dissimilarities.

**Fig 3:**
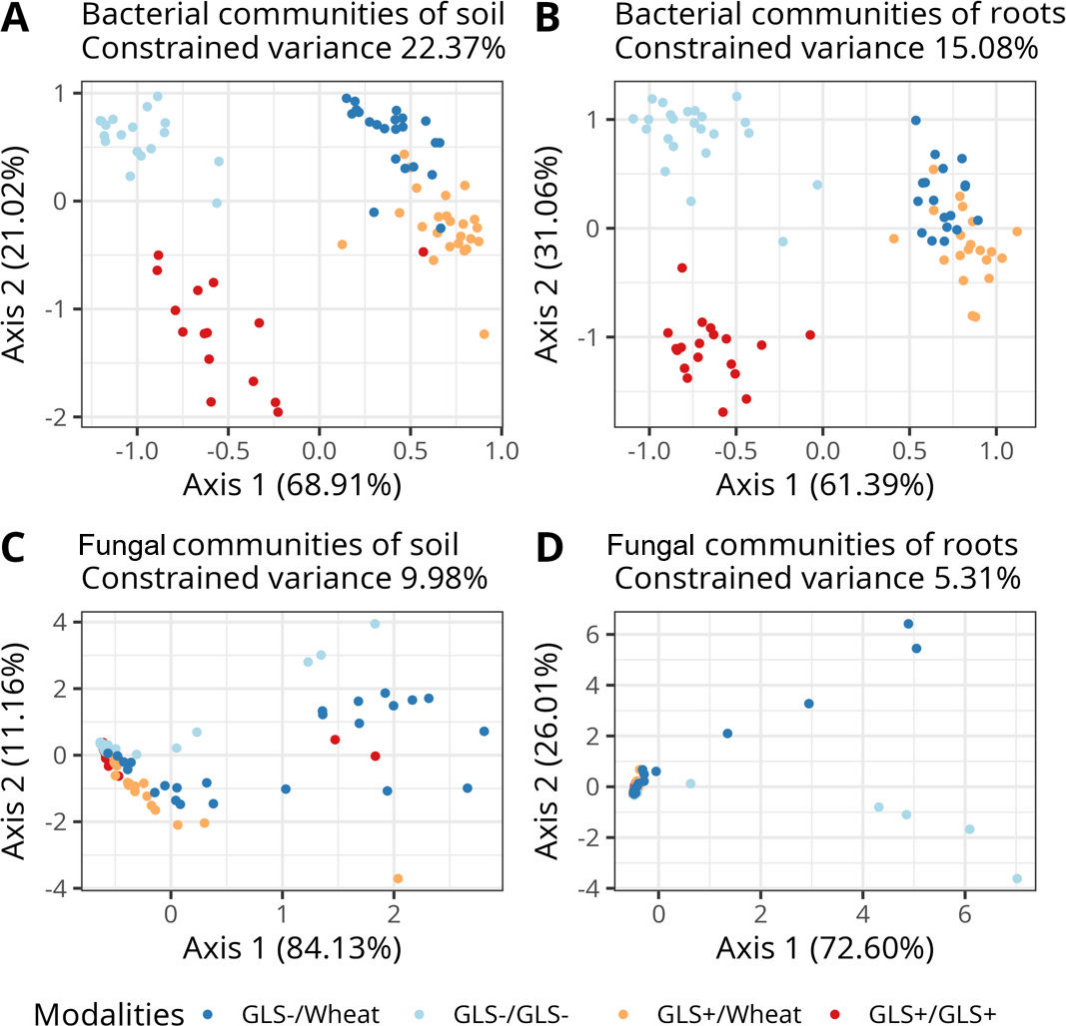
Beta diversity analyzed using db-RDA on Bray-Curtis distance on the bacterial communities of (**A**) the rhizosphere soil (**B**) the roots and the fungal communities of (**C**) the rhizospheric soil and (**D**) the roots. The four modalities tested correspond to the two rapeseed genotypes (GLS+ and GLS−) combined with soil legacy conditions (GLS−, GLS+, and Wheat) to obtain: GLS−/Wheat, GLS−/GLS−, GLS+/Wheat, and GLS+/GLS+.

For bacterial communities, the dbRDA explained 22.37% of the variance in soil (Fig. 3A) and 15.08% for roots (Fig. 3B). Community composition differed significantly among plant modalities in both soil (PERMANOVA, df = 3, *R²* = 0.18, F = 5.70, *P* < 0.001) and root compartments (PERMANOVA, df = 3, *R²* = 0.12, F = 4.17, *P* < 0.001). These results indicate that both rhizopheric soil and root-associated bacterial assemblages were strongly structured by the experimental modalities, with a larger proportion of variance explained in the soil compartment.

For fungal communities, the dbRDA explained a lower proportion of the variance, with 9.98% in soil (Fig. 3C) and 5.31% in roots (Fig. 3D). Nevertheless, PERMANOVA analyses revealed a significant difference in fungal composition among modalities in both soil (df = 3, *R²* = 0.099, F = 3.034, *P* < 0.001) and roots (df = 3, *R²* = 0.053, F = 1.623, *P* = 0.038). This suggests that although fungal communities are less affected than bacteria, they are still influenced by plant modality in both compartments.

Altogether, these analyses demonstrated that both bacterial and fungal community structures are shaped by host soil legacy and host genotype, with bacterial communities showing stronger and more compartment-specific differentiation than fungal assemblages.

### Effect of soil legacy and rapeseed genotype on larval and adult bacterial and fungal communities

To evaluate how soil legacy and plant genotype influenced insect-associated microbiomes, we analyzed the bacterial and fungal communities of both larval and adult stages of *D. radicum*. Because insect-associated micro-organisms may originate from vertically transmitted symbionts as well as from environmental acquisition, we specifically tested whether soil legacy and rapeseed genotype shaped community composition and diversity across developmental stages.

### Alpha diversity

To assess whether soil legacy and rapeseed genotype influenced the gut-associated microbiota of *D. radicum* across developmental stages, we compared bacterial and fungal diversity in larvae and adults.

In larval bacterial communities, neither richness nor diversity was significantly affected by modality. The number of observed ASVs (ANOVA, F = 1.26, df = 3, *P* = 0.29, Fig. S5A) and the Shannon Index (ANOVA, F = 1.66, df = 3, *P* = 0.18, Fig. S5C) did not differ significantly, indicating that early-stage microbial assemblages were relatively stable and not strongly shaped by soil legacy or host genotype. In contrast, adult bacterial communities exhibited clear differences: both richness (Kruskal-Wallis test, χ² = 21.76, df = 3, *P* < 0.001) and diversity (Kruskal-Wallis test, χ² = 20.8, df = 3, *P* < 0.001) were significantly higher in adults emerging from GLS+ genotype and in the G0 adults (Fig. S7B and D). This suggests that bacterial communities diversify during insect development and are shaped by the host plant environment.

Fungal communities displayed an opposite trend between life stages. In larvae, both richness and diversity were significantly higher in GLS+ genotypes (Observed ASVs: Kruskal-Wallis test, χ² = 89.69, df = 3, *P* < 0.001, Fig. S6A, Shannon Index: Kruskal-Wallis test, χ² = 30.47, df = 3, *P* < 0.001, Fig. S6C), supporting a strong influence of plant genotype on fungal colonization. In adults, fungal richness differed significantly among modalities (ANOVA, F = 3.5, df = 3, *P* = 0.005, Fig. S6B) with adults from GLS−/GLS− modality exhibiting lower richness than those from GLS+/Wheat. Similarly, fungal diversity was significantly reduced in adults from GLS−/GLS− modality compared to those from the G0, GLS+/Wheat, and GLS+/GLS+ modalities (Kruskal-Wallis test, χ² = 19.36, df = 3, *P* < 0.001, Fig. S6D).

### Composition of microbial communities

To characterize the dominant taxa within the gut microbiota of *D. radicum*, we analyzed bacterial and fungal community composition in both larvae and adults. Across all modalities, larval and adult bacterial communities were mainly dominated by *Rickettsiales* corresponding to the genus *Wolbachia* (Fig. S7). This strong prevalence suggests that vertically transmitted symbionts constitute a core component of the *D. radicum* microbiome. In contrast, fungal communities exhibited greater variability between developmental stages. Larval gut mycobiota were mainly composed of *Ascomycota* (Fig. S8A), indicating a relatively simple fungal assemblage at this stage. Adult fungal communities, however, displayed a more complex structure, including three dominant phyla: *Olpidiomycota, Ascomycota*, and *Basidiomycota* (Fig. S8B). This shift highlights that fungal colonization diversifies during insect development.

### Beta diversity

Because insect bacterial communities were dominated by *Wolbachia*, ASVs assigned to this genus were removed to better assess the contribution of environmentally acquired bacteria. After filtering, 143 ASVs remained for larvae, whereas no ASVs were retained for adults. This indicates that larval stages harbor a more diverse assemblage of bacteria potentially shaped by soil and host genotype, while adults rely primarily on their vertically transmitted symbiont.

**Fig 4:**
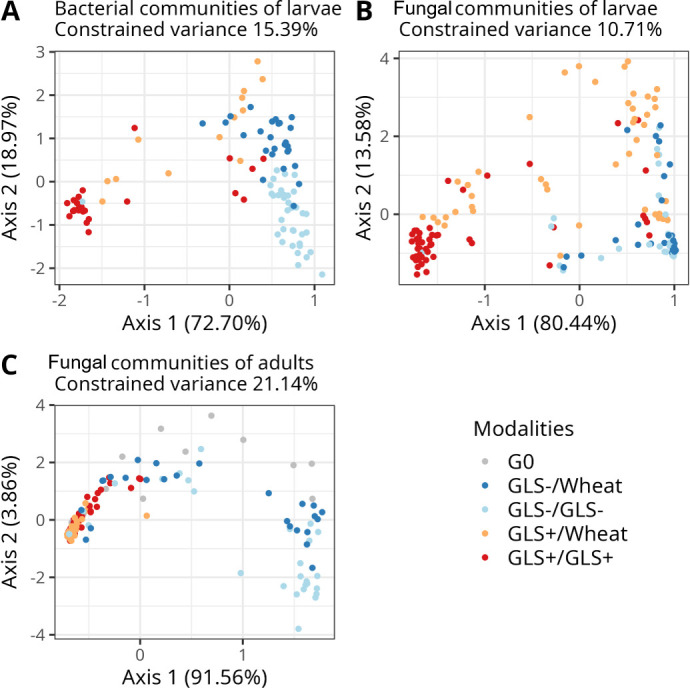
Beta diversity analyzed using db-RDA on Bray-Curtis distance on (**A**) the bacterial communities of larvae; (**B**) the fungal communities of larvae (**B**) and (**C**) the fungal communities of adults of *Delia radicum* developing on *Brassica napus* growing in different soils. The four modalities tested correspond to the two rapeseed genotypes (GLS+ and GLS−) combined with soil legacy conditions (GLS−, GLS+, and Wheat) to obtain: GLS−/Wheat, GLS−/GLS−, GLS+/Wheat, and GLS+/GLS+.

Analyses of beta diversity revealed that larval bacterial community composition was significantly influenced by both soil legacy and rapeseed genotype (PERMANOVA, df = 3, *R²* = 0.15, F = 5.27, *P* < 0.001, Fig. 4A). dbRDA based on Bray-Curtis distances showed that these factors together explained 15.39% of the total variance. This suggests that the surrounding soil environment and host genotype exert selective pressure on the subset of bacteria colonizing larvae.

For fungal communities, both larvae and adults showed significant shifts in beta diversity according to soil legacy and rapeseed genotype (larvae: PERMANOVA, df = 3, *R²* = 0.10, F = 6.63, *P* < 0.001, Fig. 4B, Adults: PERMANOVA, df = 3, *R²* = 0.21, F = 9.78, *P* < 0.001, Fig. 4C). The variance explained by dbRDA was 10.71% for larvae and 21.14% for adults, indicating that fungi are more strongly responsive to environmental factors than bacteria. These results highlight that bacterial communities were constrained by *Wolbachia* dominance in adults while fungal communities changed between larvae and adults.

### Shared ASVs between soil, roots, and larvae

To assess the potential contribution of environmental reservoirs to insect microbiota, we identified microbial taxa consistently shared across rhizospheric soil, roots, and larvae within each plant modality.

**Fig 5:**
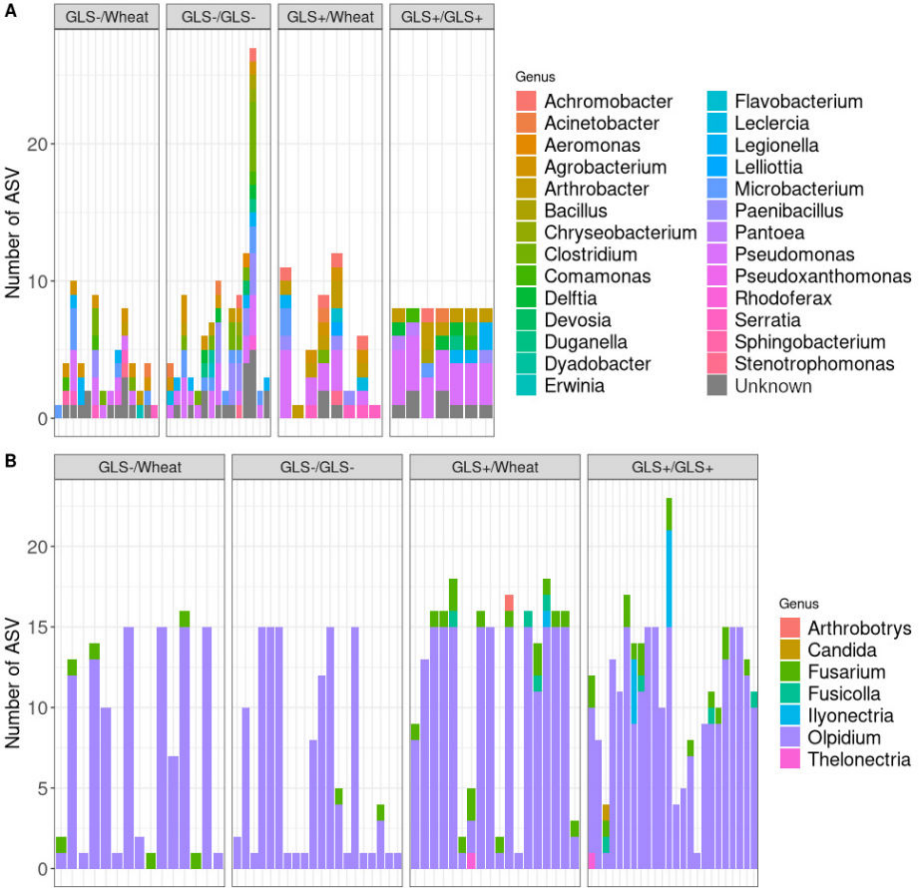
Number of bacterial (**A**) and fungal (**B**) ASVs according to the genus and shared between the rhizospheric soil, the roots, and the larvae of *Delia radicum* developing on *Brassica napus* growing in different soils. The four modalities tested correspond to the two rapeseed genotypes (GLS+ and GLS−) combined with soil legacy conditions (GLS−, GLS+, and Wheat) to obtain: GLS−/Wheat, GLS−/GLS−, GLS+/Wheat, and GLS+/GLS+. Each column on the x axis represents a plant.

For bacteria, 27 genera and 39 bacterial species overlapped soil, roots, and larvae (Fig. 5A). Among these, eight species were consistently shared across all four modalities: *Acinetobacter calcoaceticus, Arthrobacter* sp.*, Clostridium* sp.*, Microbacterium* sp.*, Paenibacillus* sp.*, Pseudomonas brassicacearum, Pseudomonas frederiksbergensis, and Pseudomonas* sp. In addition, each modality was associated with its own specific set of shared species. For instance, *Pantoea agglomerans, Bacillus circulans, and Pseudomonas kilonensis* were uniquely shared in GLS+/GLS+ plants, whereas *Pseudomonas fluorescens, Paenibacillus polymyxa, Lelliottia* sp., *and Stenotrophomonas* sp. were specific to GLS+/Wheat. Interestingly, no species were simultaneously shared between the GLS+/GLS+ and GLS+/Wheat, indicating distinct environmental filtering depending on plant genotype. In contrast, the GLS−/GLS− and GLS−/Wheat modalities supported a higher number of unique shared species, including *Acinetobacter johnsonii* and *Stenotrophomonas rhizophila*. The complete list of bacterial taxa shared for each modality is provided in Table S1.

For fungi, eight fungal species were shared across soil, roots, and larvae (Fig. 5B). Two species, *Fusarium acutatum* and *Olpidium brassicae,* were consistently detected in all modalities, highlighting their strong potential as environmentally transmitted taxa. Each plant genotype-soil legacy combination also harbored modality-specific fungi. For example, *Candida vartiovaarae* was uniquely detected in GLS+/GLS+, while *Arthrobotrys oudemansii* was specific to GLS+/Wheat. Moreover, four species (*Fusarium waltergamsii, Thelonectria* sp.*, Fusicolla septimanifiniscientiae,* and *Ilyonectria macrodidyma*) were shared in both GLS+/GLS +and GLS+/Wheat, suggesting that certain fungal taxa were more closely associated with GLS +genotype regardless of soil legacy. The complete species list for each modality is given in Table S2.

Together, these results indicate that both bacterial and fungal communities contribute to potential horizontally acquired taxa for insect larvae, but the identity of these shared taxa strongly depends on the combination of soil legacy and plant genotype.

### Cultivable gut microorganisms extracted from larvae

To further characterize the gut microbiota *D. radicum* larvae, we isolated cultivable microbial species from individuals reared on GLS+ and GLS− rapeseed genotype.

In total, 20 distinct microbial taxa were recovered from larval guts across both plant genotypes. Among these, six species were consistently found in larvae reared on GLS+ or GLS− plant genotypes: the bacteria *Achromobacter kerstersii, Pantoea agglomerans, Erwinia persicina, Stenotrophomonas* sp.*,* as well as the yeasts *Candida* sp. and *Neoascochyta exitialis*. In addition to this shared core set, clear genotype-specific differences emerged. Larvae reared on GLS plants harbored six unique bacterial species: *Brucella rhizosphaerae, Comamonas koreensis, Erwinia aphidicola, Paenibacillus odorifer, Pseudomonas zeae, and Stenotrophomonas tumulicola*. Conversely, larvae from GLS+ plants were associated with eight exclusive taxa: *Achromobacter aegrifaciens, Acinetobacter calcoaceticus, Bacillus mycoides, Siccibacter turiscensis, Klebsiella pneumoniae, Pseudomonas brassicacearum, Pseudomonas corrugata, and Pseudomonas migulae*. These findings indicate that beyond a shared microbiota, the glucosinolate content of the host plant and by extension, the ITC level may shape the composition of the larval gut community by favoring or excluding specific microbial taxa.

### PEITC tolerance of cultivable gut microorganisms

**Fig 6:**
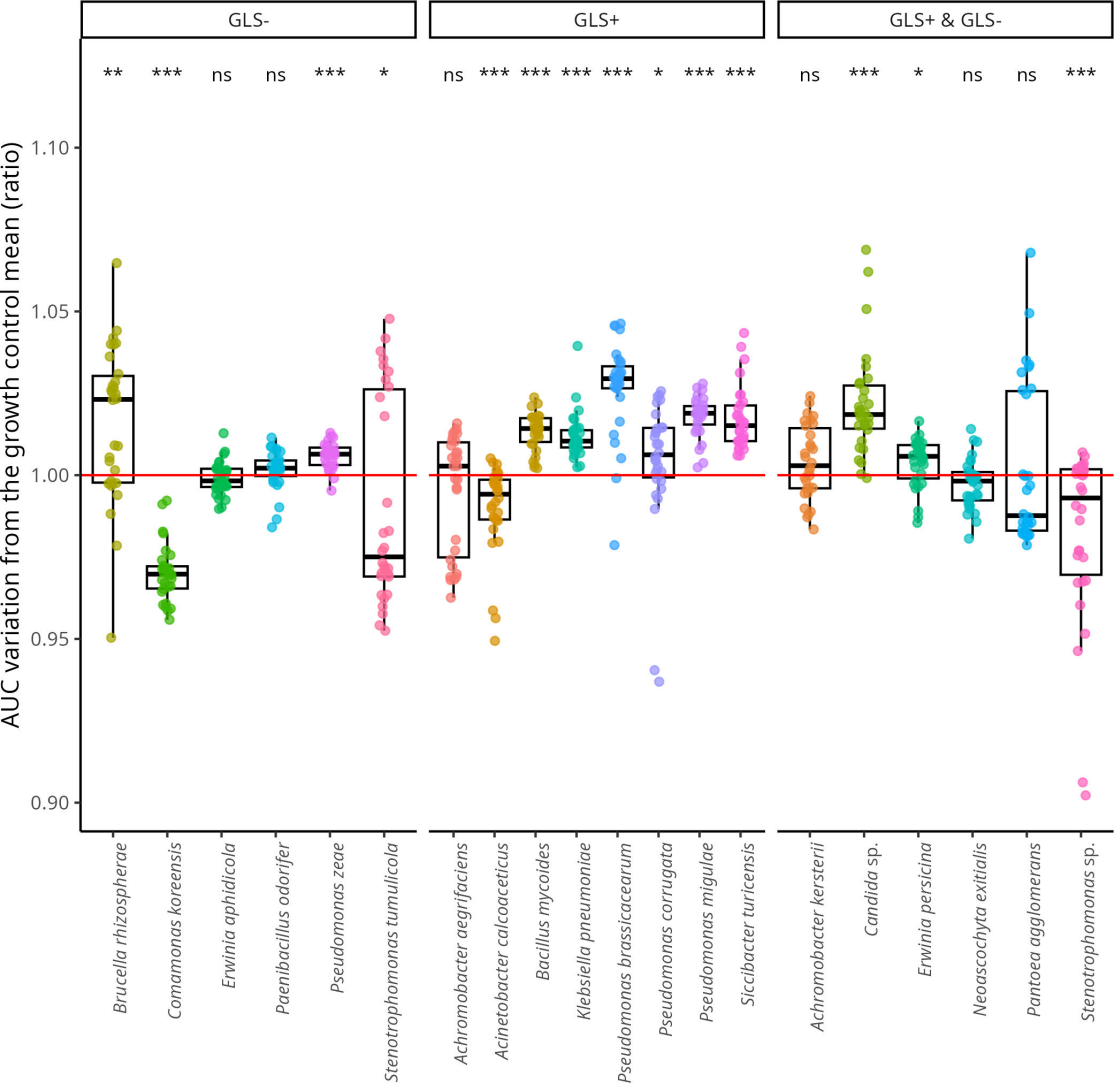
Relative growth (area under the curve, AUC) of 20 bacterial isolates at 125 µM of PEITC compared to PEITC-free control. The red line corresponds to the similar growth in both control and PEITC treatments (*i.e*., ratio = 1). Values above 1 indicate a higher AUC than the control mean, while values below 1 indicate a reduced AUC. Mann-Whitney symbols indicate paired Whitney tests performed between the control treatment (PEITC concentration = 0 µM) and the PEITC treatment (ns = not significant, * =*P* < 0.05, ** =*P* < 0.01, *** =*P* < 0.001).

To assess the PEITC tolerance of the microorganisms isolated from larval guts, we conducted growth assays in a millifluidic system in the absence and presence of PEITC (125 µM). We monitored the growth of 20 isolates obtained from GLS+, GLS−, and GLS+/GLS− larvae. To determine whether the presence of PEITC can affect the growth of these bacterial isolates, we compared the lag time, the midpoint, and the area under the curve (AUC) between PEITC-treated and control conditions (see methods for parameter selection). The AUC differed significantly between conditions without PEITC and with 125 µM PEITC for fourteen species (Fig. 6; Fig. S11). A significant increase in AUC was observed for *Pseudomonas brassicacearum* (Mann-Whitney, U = 834, *n* = 30, *P* < 0.001), *Pseudomonas migulae* (Mann-Whitney, U = 882, *n* = 30, *P* < 0.001) and *Siccibacter turicensis* (Mann-Whitney, U = 816, *n* = 30, *P* < 0.001), indicating higher overall growth in the presence of PEITC. In contrast, *Comamonas koreensis* (Mann-Whitney, U = 15, *n* = 30, *P* < 0.001), *Acinetobacter calcoaceticus* (Mann-Whitney, U = 205, *n* = 30, *P* < 0.001), and *Stenotrophomonas* sp. (Mann-Whitney, U = 186, *n* = 30, *P* < 0.001) showed reduced AUC values, reflecting increased sensitivity to PEITC.

Lag time analyses confirmed contrasted responses between species (Fig. S9 ans S12). The most pronounced effect was observed for *Paenibacillus odorifer* (Mann-Whitney, U = 841, *n* = 30, *P* < 0.001), *Bacillus mycoides* (Mann-Whitney, U = 411, *n* = 30, *P* < 0.001), and *Pseudomonas brassicacearum* (Mann-Whitney, U = 697, *n* = 30, *P* < 0.001), which exhibited significantly longer lag time in the presence of PEITC.

Midpoint values also varied significantly between treatments for nine species (Fig. S10 and S13). In *Pseudomonas brassicacearum* (Mann-Whitney, U = 674, *n* = 30, *P* < 0.001) and *Stenotrophomonas* sp. (Mann-Whitney, U = 768, *n* = 30, *P* < 0.001), the midpoint was significantly higher under PEITC, reflecting delayed exponential growth. Conversely, *Erwinia aphidicola* showed lower midpoints values (Mann-Whitney, U = 218, *n* = 30, *P* < 0.001, suggesting faster entry into the exponential growth phase despite PEITC exposure.

Overall, these results highlight that microbial isolates respond differently to the presence of PEITC, resulting in contrasting growth dynamics. Some species undergo an initial growth delay followed by recovery or increased final biomass, whereas others show persistent inhibition across all growth phases. These findings suggest that PEITC strongly influences microbial growth kinetics, consolidating its potential role in microbial community assembly.

### Detection of ITC-hydrolase gene *saxA* in cultivable gut microorganisms

To investigate whether gut-associated microorganisms possess the genetic capacity to degrade GLS, we screened all cultivable isolates for the presence of the *saxA* gene by PCR amplification and sequencing. Among the 20 isolated species, *Pseudomonas brassicacearum* was the only taxon in which *saxA* was detected. This finding is particularly relevant because *P. brassicacearum* was also consistently identified in the metabarcoding data set and occurred across larvae, soil, and root compartments, suggesting a dual association with the host and the environment.

**Fig 7:**
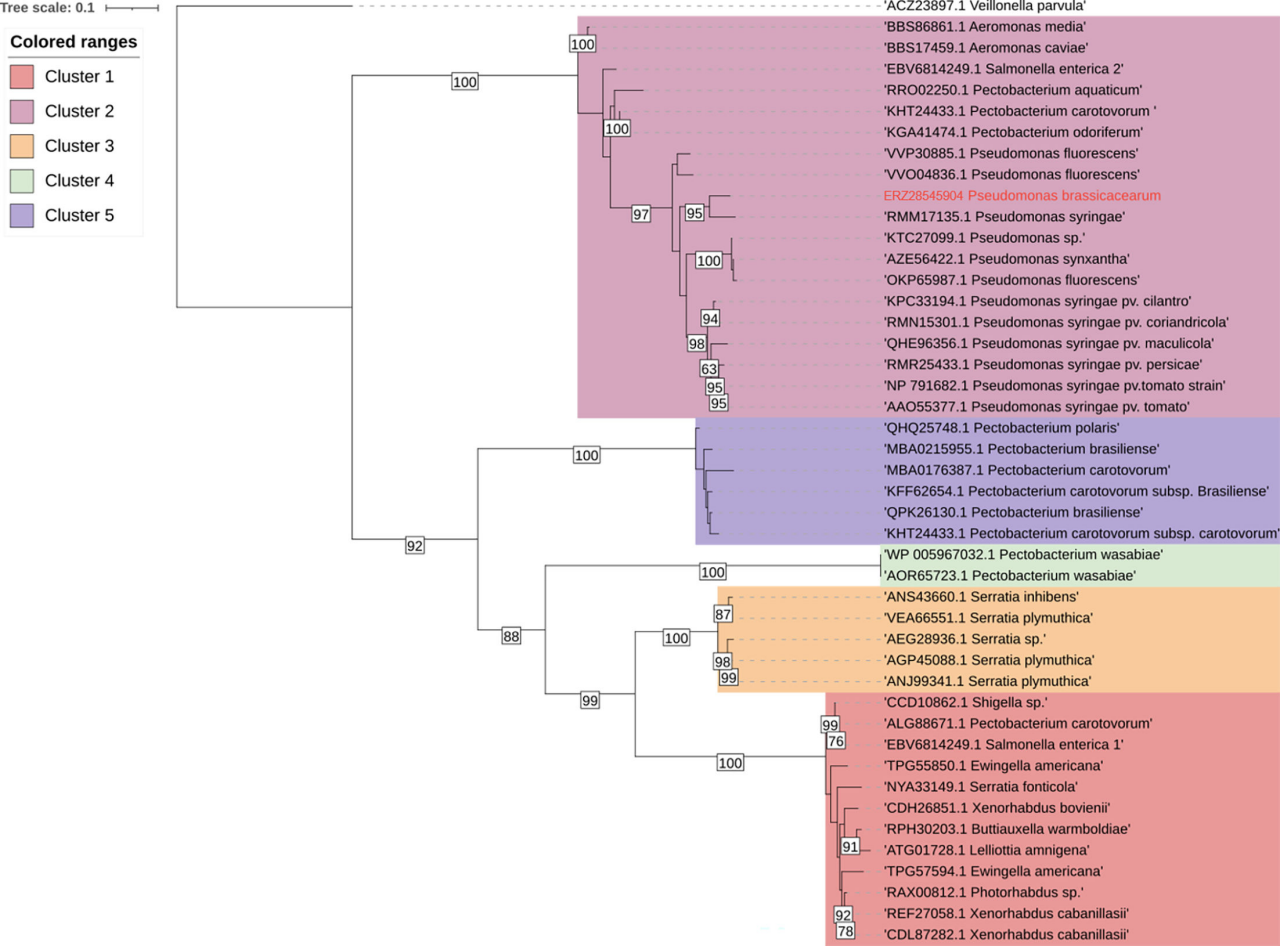
Maximum-likelihood tree of *saxA* homologs from the RefSeq database. DNA sequences were aligned with MAFFT, and phylogenetic trees were calculated with RAxML using 1,000 bootstraps. Only bootstrap values above 60 are indicated.

To place this gene into an evolutionary context, we performed a phylogenetic reconstruction of *saxA* homologs, using a maximum-likelihood approach. The analysis revealed five distinct clusters (Fig. 7), with the *P. brassicacearum* sequence grouping within Cluster 2, alongside homologs from *Pseudomonas*, *Aeromonas*, and *Pectobacterium*. These results suggest that *P. brassicacearum* may be able to degrade ITCs.

### Functional assay of PEITC degradation by *Pseudomonas brassicacearum*

**Fig 8:**
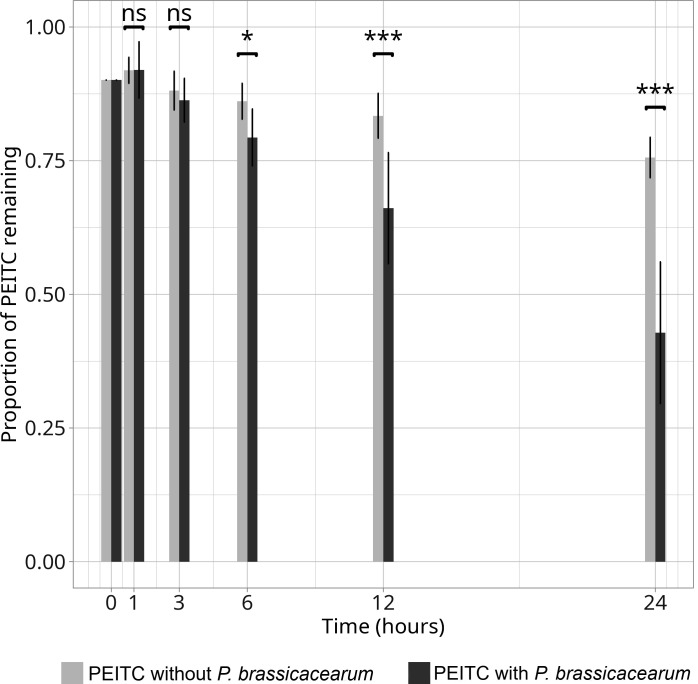
Proportion of PEITC remaining in the media with (dark gray) and without (light gray) *Pseudomonas brassicacearum* at 0 h, 1 h, 3 h, 6 h, 12 h, and 24 h after its addition to the medium. ANOVA, ns: *P* > 0.05, *: *P* < 0.05, ***: *P* < 0.001.

To assess whether *P. brassicacearum* could detoxify PEITC, we monitored its concentration over time in culture media with or without *P. brassicacearum*. The results showed a marked decrease in PEITC concentration in the presence of the bacterium (Fig. 8). Statistical analysis revealed that the decline in PEITC was significantly greater in media containing *P. brassicacearum* compared with control media starting at 6 hours, with stronger contrasts at 12 hours and 24 hours (ANOVA, χ² = 46.5, df = 5, *P* < 0.001). These findings suggest that *P. brassicacearum* is able to degrade PEITC, suggesting that this gut-associated bacterium can contribute to the detoxification of GLS breakdown products.

### Morphometric measurements of larvae and adults

To evaluate the performance of *D. radicum* on different genotypes and soil legacies, we measured hatching rates, larval and adult abundance per plant, and larval performance traits.

**Fig 9:**
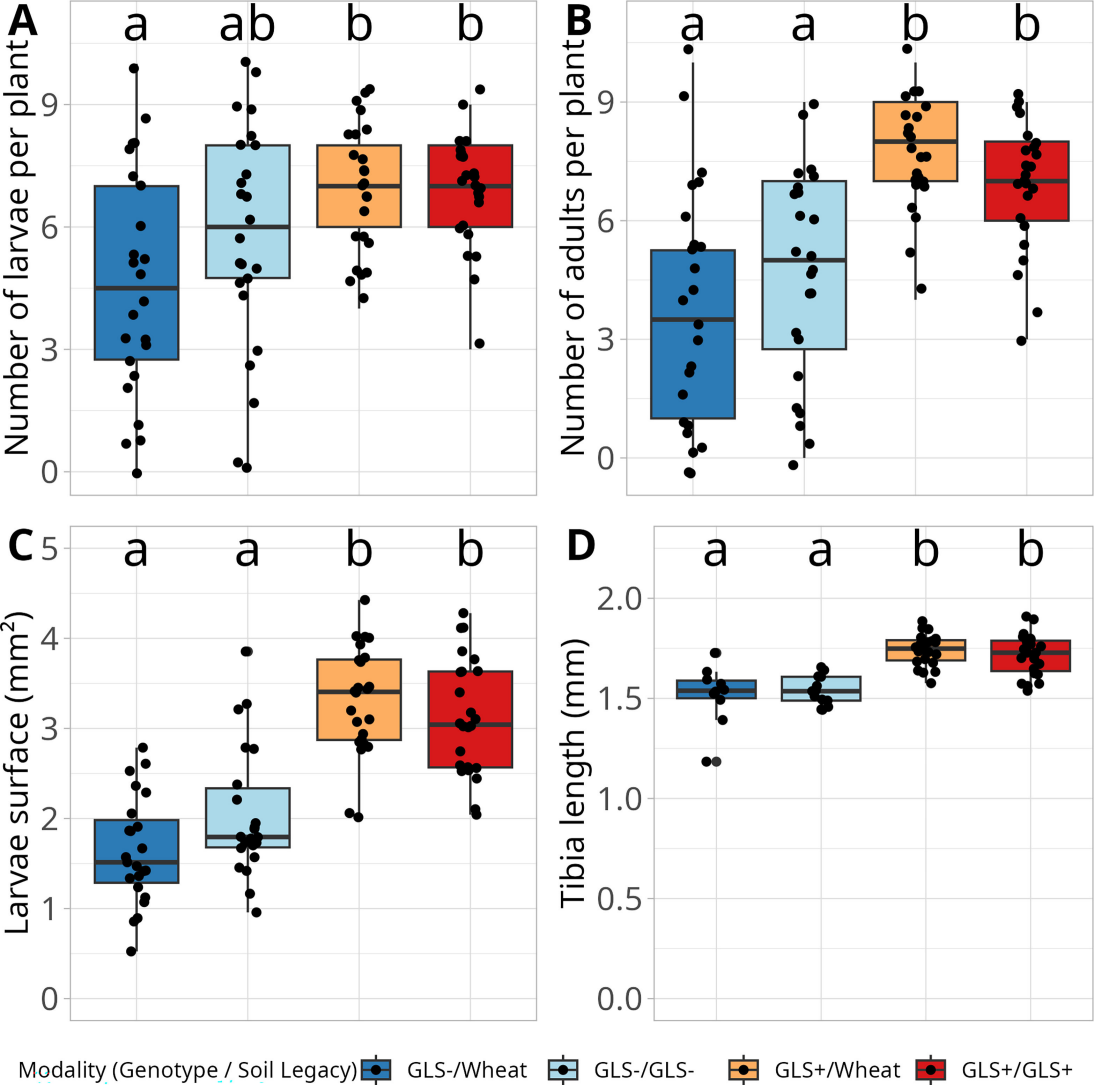
Survival and morphometric parameters of *Delia radicum* developing on *Brassica napus* growing in different soils. The four modalities tested correspond to the two rapeseed genotypes (GLS+ and GLS−) combined with soil legacy conditions (GLS−, GLS+, and Wheat) to obtain GLS−/Wheat, GLS−/GLS−, GLS+/Wheat, and GLS+/GLS+. (**A**) Number of larvae per plant; (**B**) number of adults emerging from each plant; (**C**) surface of collected larvae; (**D**) tibial length of emerging flies. ANOVA, Different letters indicate a significant difference, *P* < 0.05.

The hatching rate measured in Petri dishes was 68%, confirming egg viability under laboratory conditions. On plants, larvae were significantly more abundant in GLS +modalities compared to the GLS−/Wheat (ANOVA, F = 5.89, df = 3, *P* < 0.001, Fig. 9A), and the number of emerging adults per plant (ANOVA, F = 15.7, df = 3, *P* < 0.001, Fig. 9B) was also significantly higher. Additionally, larval surface and tibia length were significantly greater in GLS +modalities (larval surface: ANOVA, F = 37.22, df = 3, *P* < 0.001, Fig. 9C, tibia length: ANOVA, F = 21.50, df = 3, *P* < 0.001, Fig. 9D). These results indicate that GLS +plants supported higher larval growth and survival, suggesting that the host plant genotype strongly influences insect performance and development.

## DISCUSSION

By combining an experimental design incorporating distinct plant and *B. napus* genotypes differing in glucosinolate (GLS) content, we demonstrated that the composition and structure of microbial communities are strongly influenced by environmental conditions. Likewise, the structure of the microbial communities associated with *D. radicum* larvae was primarily shaped by the host plant genotype. Our metabarcoding-based approach highlights microbial overlap across compartments, with several taxa shared between the rhizosphere, the roots, and the insect larvae. Complementing this, a culture-based approach allowed the isolation of twenty microbial species from the larval gut of *D. radicum*. PCR screening and sequencing identified *Pseudomonas brassicacearum* as the sole bacterium harboring the *saxA* gene, a key gene involved in isothiocyanate (ITC) detoxification. Notably, this species was also detected in both the insect and its surrounding environment. Functional assays confirmed the ability of *P. brassicacearum* to degrade the 2-phenylethyl isothiocyanate (PEITC), a toxic GLS-derived compound. In addition, gut-derived microbial isolates exhibited a heterogeneous response to PEITC, ranging from growth inhibition to increased growth or growth recovery after a prolonged lag phase. Finally, our morphometric and survival analysis showed that *D. radicum* exhibited enhanced performance on GLS+ plants, suggesting an adaptive benefit mediated by the microbiota and its capacity to mitigate the effects of plant chemical defenses.

### Microbial communities differ significantly between the rhizosphere and *Brassica napus* roots, reflecting plant genotype-specific selection pressures

The composition and structure of microbial communities differed between the rhizospheric soil and *B. napus* roots, consistent with previous studies (37, 38). As previously reported, the GLS+ genotype exhibited higher concentrations and greater compositional diversity of GLS in root tissues compared to the GLS− genotype.

Our experimental design demonstrated that microbial diversity can be shaped through selective pressure toward the enrichment of ITC-degrading microbial functions in the rhizosphere. From a holobiont perspective, we hypothesize that microorganisms with ITC-degrading capabilities present in the rhizosphere may be horizontally transmitted to the insect host, thereby potentially influencing its physiological performance (39, 40).

In the field, crop succession significantly influenced the microbial community composition. Bacterial richness and diversity in the rhizospheric soil were reduced in the GLS+/GLS +modality, suggesting that continuous monoculture may exert selective pressure that diminishes overall bacterial diversity. This trend aligns with findings from monoculture wheat systems, where a decline in rhizospheric bacterial diversity following repeated wheat cropping was reported (41). In contrast, monoculture conditions may promote the proliferation of plant pathogens or bacteria adapted to the specific soil environment shaped by the repeated cultivation of a single crop (42).

This was evident in our study, where fungal communities were predominantly composed of *Olpidium brassicae* in the rhizospheric soil under both GLS+ and GLS– rapeseed successions, as well as in the roots of both genotypes. *O. brassicae* is a known endophytic fungus associated with the rhizosphere of rapeseed and was detected in high abundance following rapeseed crop successions (43). This species has also been reported as dominant in the rhizosphere of various *B. napus* genotypes (44), particularly under monoculture systems (45).

### *Wolbachia* vertical transmission and horizontal acquisition of plant genotype-specific microbiota

The bacterial communities observed in *D. radicum* larvae and adults were dominated by the endosymbiont *Wolbachia*. This intracellular bacterium commonly exhibits high infection prevalence in *D. radicum* populations, reaching up to 65% (27) and accounting for up to 80% of amplicon sequence variants (ASVs) in other studies (28, 30). *Wolbachia* has also been shown to reduce the overall diversity of insect-associated bacterial communities, as demonstrated in both *D. radicum* (30) and *Drosophila melanogaster* (46). In our study, Wolbachia was the only bacterial species found to be vertically transmitted in *D. radicum*.

Not all microbial genera detected in the rhizospheric soil and roots were present in the *D. radicum* gut microbiota. This divergence likely results from the insect gut environment acting as a selective filter, promoting certain microbial taxa while excluding others. This selective filtering is governed by a complex interplay of factors, including gut pH, oxygen availability, nutrient composition, and the host immune response (47).

We hypothesized that horizontal transmission between the environment and the larvae would be influenced by the interaction between plant genotype and soil legacy. Supporting this, certain bacterial genera such as *Erwinia*, *Sphingomonas*, and *Rhodoferax* were horizontally transmitted in the GLS– context, whereas *Pantoea* was specifically transmitted in the GLS+ context. This suggests that GLS concentration—whether high or low—plays a crucial role in shaping the larval microbiome through differential horizontal acquisition. In the GLS+ context, the specific transmission of *Pantoea* may reflect its potential adaptation to GLS compounds or their degradation products, as observed in flea beetle gut isolates (25).

Other bacterial genera such as *Pseudomonas*, *Paenibacillus*, *Clostridium*, and *Microbacterium* were shared across environmental contexts, indicating that they may be less sensitive to variations in GLS concentration.

Taxonomic identification alone does not provide insights into the functional roles of horizontally transmitted microbes. In this study, the cultivation of gut microorganisms from *D. radicum* allowed us to circumvent the dominance of nonculturable *Wolbachia* and to specifically focus on the functional capacity of gut bacteria to degrade isothiocyanates (ITC).

### PEITC tolerance of cultivable gut microorganisms

All gut-associated bacterial isolates from *Delia radicum* grew in the presence of 125 µM PEITC, but responses varied among species: for example, *Achromobacter kersterii* and *A. aegrifaciens* showed no significant change in growth, *Pseudomonas* species (*P. brassicacearum*, *P. migulae*) exhibited delayed lag phases, while *Comamonas koreensis* and *Acinetobacter calcoaceticus* displayed an overall growth inhibition.

Our results indicate that PEITC likely shapes the gut microbiome composition and dynamics by modulating the growth of individual taxa. While tolerant species will be favored by high PEITC content, PEITC-degrading species may enable sensitive taxa to persist. Similar strain-specific heterogeneity in tolerance to plant secondary metabolites has been reported in the honey bee gut microbiota, where only certain bacterial isolates were able to degrade amygdalin, and where the underlying mechanisms varied between strains (48). Comparable patterns were observed in termite gut symbionts, in which some isolates showed tolerance to imidacloprid, an insecticide, while others remained sensitive, highlighting the strong selective pressure imposed by toxic compounds (49).

The observed differences in growth dynamics suggest the activation of resistance or detoxification mechanisms, potentially imposing physiological costs such as extended lag phases. For example, microbial defense mechanisms, including the production of exopolysaccharides, have been documented in *Pseudomonas aeruginosa* and *Staphylococcus aureus* and can reduce bacterial growth (50, 51). In addition, these observations corroborate studies in the literature that have shown that the production of β-lactamases such as *AmpC* has a metabolic cost for *Pseudomonas aeruginosa* without being a significant biological disadvantage (52).

To identify the molecular mechanisms of PEITC tolerance and detoxification, future work combining transcriptomics and metabolomics approaches would be required. Moreover, linking microbial detoxification capacity to larval performance would clarify the ecological relevance of these interactions. Achieving this would require the development of a gnotobiotic system for *D. radicum*, which is currently unavailable due to the complexity of rearing larvae on artificial media (53). A synthetic microbiome approach using such a gnotobiotic system would allow for directly testing whether the gut microbiota can bolster host tolerance to GLS breakdown products.

### Detoxification function of *Delia radicum* gut bacteria

We hypothesized differential prevalence of ITC degradation genes among gut bacteria isolates and found *P. brassicacearum* to be the sole carrier of the *saxA* gene and to be able to degrade PEITC *in vitro*. The presence of the *saxA* gene and the ability to degrade ITC *in vitro* has been shown in other species such as *Pseudomonas syringae* pv. *tomato* (54) and *Pectobacterium* sp. (55), which are phytopathogenic bacteria. *saxA* is a virulence factor that can overcome chemical defense systems in Brassicaceae (56). In our study, *P. brassicacearum* was able to degrade PEITC *in vitro* and carried the *saxA* gene in an ITC-rich environment. This could suggest that the expression of this gene is the factor that enables the bacterium to overcome the plant chemical defenses present in the larval gut.

A previous study found four isolates from the gut of *D. radicum* harboring a plasmid that encodes a *saxA* homolog (*Pectobacterium* sp., *Acinetobacter* sp., *Serratia* sp. and *Providencia* sp.) (31). However, we had no species in common in our study that carries *saxA*, suggesting that there is no relationship between taxonomy and *saxA* homolog prevalence. *saxA* has been shown to be carried by a plasmid in *Pectobacterium* (31). Subsequent transfer of this plasmid into *E. coli* conferred the ability to degrade PEITC. Furthermore, when *saxA* was expressed in a *Pectobacterium parmentieri* strain, which does not naturally carry the gene, it increased the strain’s ability to macerate *Arabidopsis thaliana* (56).

Herein and in another study (31), different bacteria without *saxA* were isolated from the gut of *D. radicum*. One hypothesis is that alternative detoxification mechanisms, independent of *saxA*, may be employed by plant- and gut-associated microbes to survive in ITC-rich environments. For example, resistance mechanisms such as biofilm formation, enzymatic inhibition, or efflux pumps, which are known mechanisms in antibiotic resistance (57), could be used by these bacteria to cope with ITC.

These findings highlight the diversity of microbial detoxification strategies. Future investigations employing functional approaches could further elucidate the detoxification strategies of microbes confronted with plant specialized metabolites.

### Enhanced performance of *D. radicum* on GLS+ plants

Insect survival, larval surface, and tibia length were greater in *D. radicum* developing on GLS+ plants. These parameters, often used as proxies for insect fitness (58), indicate a better performance of *D. radicum* when the genotype is characterized by a high concentration of GLS. This result is rather counterintuitive at first sight because the plants with the highest concentration of toxic compounds induce a better insect performance. In generalist species, such as *Spodoptera littoralis* and *Mamestra brassicae*, GLS negatively affected the development of larvae (59). However, in the specialist lepidopteran species, *Pieris rape*, GLS had a positive effect on oviposition and larval survival (60). Therefore, there may be a difference between specialist and generalist herbivorous species in their adaptation to GLS, with the former having a better performance. The response to root herbivory in brassicaceous plants and isothiocyanate detoxification in *D. radicum* and *D. floralis* has been addressed (24, 61), but because both species are specialists feeding on *Brassica* roots, more research is required to understand how generalists and specialists deal with *Brassica* toxic compounds. Nevertheless, ITC reduced *D. radicum* performance, and low and high genotypes of *B. rapa* had the same activation of indole biosynthesis genes when infested by *D. radicum* (24, 61). Consequently, these results do not provide an explanation for the differences in performance according to the plant GLS content found in our study. GLS are, however, detected by the flies and play a role in the oviposition of insects (62). More research is needed on the consequence of GLS on larval feeding and survival.

Aside from the GLS content, this result could also be explained by differences in the nutritive value between the two genotypes tested. Our results showed a higher carbohydrate content only when a GLS+ genotype was grown on a GLS+ Soil legacy, while there was no difference for the protein concentration. Consequently, differences in nutritive value alone cannot explain the differences in insect fitness. In our experiment, we determined the GLS concentration in our plants, as a proxy for ITC produced, but other potential toxic compounds remain to be identified. Indeed, Brassicaceae produce other toxic compounds such as flavonoids and saponins, which have antifeedant or insecticidal properties on insects (63). Alternatively, testing other GLS genotypes, and several GLS + genotypes in particular, would provide insights into the interplay between primary and specialized metabolites and its influence on *D. radicum* fitness.

### Perspective

The complementary approach based on metabarcoding of the *D. radicum* gut and the culture and isolation of microbiota conducted herein detected a species, *P. brassicacearum*, harboring the ITC detoxifying gene *saxA*. However, our approach alone did not quantify the abundance of *saxA* in the gut of *D. radicum* or in the fly environment. New tools based on quantification of *saxA*, using, for example, quantitative PCR, and potential *saxA* expression would be required to achieve this purpose. Such tools would allow us to test the hypothesis that our experimental design actually represented a gradient of *saxA*. More generally, quantification of *saxA* would also determine whether the bacterial community would provide a fundamental function of ITC detoxification in the *D. radicum* gut or whether this role is only secondary.

In this work, we focused on the detoxification by the gut microbiota, but this function is also carried out by the insect through the mercapturic acid pathway and an ITC-specific hydrolytic pathway. Characterizing detoxifying mechanisms through the microbiota as well as the insect physiology altogether would give a more exhaustive picture of the ITC detoxification in *D. radicum* and ultimately a better understanding of the advantages and the consequences of these detoxification pathways on the insect’s overall performance. Finally, detoxification through *saxA* may only be one of the important functions provided to the insect by its microbiota. Identifying other functions, through a synthetic community approach (64), would provide a better understanding of the role the microbiota plays in *Brassica-D. radicum* interactions.

## MATERIALS AND METHODS

### Effects of rapeseed genotype and soil legacy on microbiota and *Delia radicum* performance

#### Plant-mediated microbial soil legacy

Soil used in this experiment was collected from INRAE Le Rheu experimental field on 24 August 2020 (La Gruche, Le Rheu, France, 48°08’22”N 01°48’05”W). To generate a gradient of presumed abundance of bacteria capable of degrading isothiocyanates (ITC), two *B. napus* genotypes were selected: Romeo, a high GLS content genotype (44 287.9 nmol.g^−1^ roots dry weight) and Bronowski, a low GLS content genotype (5 965.5 nmol.g^−1^ roots dry weight) (32). In 2021, three distinct plant-mediated microbial soil legacy treatments were established through the cultivation of different plants over three successive 5-week cycles:

- *Triticum aestivum* var Atlas-mediated microbial soil legacy: named Wheat, a control modality without GLS
- *Brassica napus* cv Bronowski-mediated microbial soil legacy: named GLS− with low GLS content
- *Brassica napus* cv Romeo-mediated microbial soil legacy: named GLS+ with high GLS content

At the end of each plant cycle, the soil was collected, homogenized, and reused for the next cultivation cycle, thereby applying plant-mediated microbial soil legacy selective pressure (65) and reinforcing legacy effects.

#### Cabbage root fly (*Delia radicum*) rearing

The initial *D. radicum* population was collected in 2016 from an agricultural field in Pleumeur-Gautier (Brittany, France). In the laboratory, flies were reared according to the protocol established by Neveu (66), using rutabaga (*Brassica napus subsp. rapifera*) as the host plant. Rearing was conducted in a climate-controlled chamber at 16:8 L:D, 20:18±1°C and 60 ± 10% RH. Adults from this rearing, referred to as the G0 generation, were used to produce eggs for experimental infestations. Twelve G0 females and 12 G0 males were collected and preserved in 96% ethanol at 4°C for subsequent metabarcoding analysis.

### Plant cultivation and infestation protocol

For the experimental setup, four combinations of modalities were established by crossing the two *B. napus* genotypes (GLS− or GLS+) with soil legacy conditions to obtain GLS−/Wheat, GLS−/GLS−, GLS+/Wheat, and GLS+/GLS+. A total of 192 rapeseed plants were cultivated (48 per modality), randomized into two blocks within a climate-controlled chamber at 16:8 h L:D cycle, at 20±1°C during the day and 18±1°C at night. Five weeks after sowing, each plant was infested with 10 *D. radicum* eggs that were deposited on the plant collar. To evaluate the egg hatching rate, 10 eggs were placed in Petri dishes and monitored daily for 7 days. Thirty replicates of hatching tests were conducted to quantify egg viability.

### Larvae, soil, roots, and adults sampling

Two sampling time points were selected based on the developmental timeline of *D. radicum*: the larval stage (15 days post-infestation) and the adult emergence stage (40 days post-infestation). These stages were chosen to capture the dynamics of gut microbiota across metamorphosis. At the larval stage, samples were collected from 96 plants (24 per modality) including soil, *B. napus* roots, and *D. radicum* larvae. At the adult emergence stage, flies were collected daily until emergence ceased, and a total of 96 plants were sampled.

### GLS, sugar, and protein content analysis

To validate that the selected genotypes correspond to contrasting GLS concentrations, we quantified GLS levels in the roots of *B. napus* from both genotypes (GLS− and GLS+) grown in soils with different plant-mediated microbial soil legacies (GLS−, GLS+, or Wheat).

Sample preparation protocol was adapted from Glauser et al. (67) with some modifications. Briefly, 10 mg of lyophilized root powder was suspended in 1 mL methanol and 0.1% formic acid (vol/vol). Samples were vortexed and then sonicated for 5 minutes. They were then briefly centrifuged, and the supernatant was filtered (0.45 µm) into appropriate vials before analysis.

UHPLC-MS analyses were performed on a Waters Acquity UPLC (Milford, MA) interfaced to a Waters Xevo TQD with electrospray ionization. Chromatographic separation was carried out on a CSH C18 150 × 2.1 mm, 1.7 µm column (Waters) maintained at 40°C under the elution conditions described in Table S3.

Injection volume was 2 µL. GLS were identified based on retention time, m/z ratio using a set of pure analytical standards as reference. Quantification was performed through external calibration prepared with GLS analytical standards with five points ranging from 100 ppb to 10 ppm. The GLS analytical standards mix used for quantification is described in Table S4.

Additionally, we measured soluble protein and digestible carbohydrate contents in the roots according to Deans et al. (68) protocol.

Statistical analyses were conducted with R version 4.4.3. Differences in GLS concentrations, soluble proteins, and digestible carbohydrates among modalities were compared with Kruskal-Wallis rank sum tests followed by Dunn’s post hoc test. P-values were adjusted using Bonferroni correction for multiple comparisons.

### Morphometric analysis of *D. radicum*

Length and diameter of *D. radicum* larvae were measured using a Zeiss SteREO Discovery V12 binocular microscope in combination with Histolab software (Microvision Instruments, Evry, France). Larval surface area was estimated from these measurements as a proxy for body size. Length of the left hind tibia of adults was also measured and served as a proxy for individual performance (58).

To evaluate the effects of *B. napus* genotype and soil legacy on *D. radicum* performance (larval and adult stages), linear models were employed. Model significance was assessed using Type II ANOVA (F-tests) with the car package (69). *Post hoc* comparisons of estimated marginal means were conducted with the Bonferroni correction for p-values, using the emmeans package (70).

### DNA extraction

Two larvae per plant were sampled for microbiota analysis. Surfaces of larvae and adults were disinfected following the protocol described by Wallinger et al. (71). DNA was extracted from soil, roots, larvae, and adults using the DNeasy 96 PowerSoil Pro Kit (Qiagen, Courtaboeuf, France) following the manufacturer’s instructions. DNA quality and concentration were assessed using a NanoDrop spectrophotometer (Thermo Fisher Scientific, Waltham, MA, USA).

### Amplicon library construction and sequencing

All extracted DNA was normalized to approximately 10 ng/µL to ensure similar PCR amplifications. To characterize bacterial communities, the gyrB gene region (encoding the B subunit of DNA gyrase) was amplified using primer pairs gyrB_aF64/gyrB_aR353 (72), which provides higher taxonomic resolution at the species level than the 16S rRNA gene (73), which was a priority for this study. Fungal communities were characterized by amplifying the internal transcribed spacer (ITS) region using ITS1F and ITS2R primers (74). Primer sequences and PCR conditions are provided in Table S5. Sequencing was performed on an Illumina MiSeq platform using the MiSeq V3 reagent kit (2 × 300 bp) at the EcogenO platform (OSUR, Rennes, France). After sequencing, the mean of raw reads was 26,091.58 and 22,081.75 reads per sample for the gyrB and the ITS targets, respectively.

### Bioinformatic and statistical analysis

Bioinformatic analysis was conducted using R version 4.4.3. Raw sequencing reads were processed using the DADA2 pipeline (v1.26, 75). For gyrB sequences, forward and reverse reads were truncated at 200 bp and 160 bp, respectively. Taxonomic classification was performed using a custom reference database (76) for gyrB-derived ASV (Amplicon Sequence Variant) and UNITE v8.3 (77) for ITS-derived ASV. Sequences corresponding to *parE* (a *gyrB* paralog gene) were filtered out. Samples with zero remaining reads were removed from the data set.

The data were further curated with the phyloseq package (78). ASVs without taxonomic assignment at the kingdom or phylum level were removed. Rare ASVs representing less than 0.1% of the total reads were also excluded to reduce noise from potential sequencing errors. Rarefaction was performed using the ranapaca package (79) with a threshold of 5,000 reads per sample to ensure inclusion of all ASVs observed at least once (80).

Alpha diversity metrics (observed ASVs richness and the Shannon Index) were calculated using the phyloseq estimate_richness function. Normal distribution of residuals was tested. Differences in alpha diversity across modalities were assessed with Kruskal-Wallis rank sum tests, followed by Dunn’s post hoc test in the case of a non-normal distribution. In case of a normal distribution of residuals, differences in alpha diversity across modalities were assessed with ANOVA, followed by pairwise comparisons made using the emmeans function in the car package. P-values were adjusted using Bonferroni correction for multiple comparisons.

Beta diversity was assessed using a distance-based redundancy analysis (dbRDA) on Bray-Curtis dissimilarities, using the vegan package (v2.6-4 [81]). Statistical significance of community structure differences among modalities was tested using PERMANOVA (999 permutations) via the adonis2 function, followed by pairwise comparison between modalities using the pairwise.perm.manova function.

To explore taxonomic overlap between compartments, we identified ASVs shared among rhizospheric soil, roots, and larvae of the same plant.

### ITC degradation function of *D. radicum* gut

#### Isolation and culture of microorganisms from the gut of *D. radicum*

In a second experiment, *D. radicum* larvae were reared on *B. napus* cv Romeo (GLS+) and *B. napus* cv Bronowski (GLS−) as in Section 2.1.2. Soil for this experiment was collected from the INRAE Le Rheu experimental field in September 2023, at the same location as in 2.1.1. Five plants of each genotype were infested with 10 eggs. Larvae were collected 15 days post-infestation. To avoid contamination by external microorganisms, larvae were disinfected following the protocol described by Wallinger et al. (65). Guts of *D. radicum* larvae were then dissected, pooled by replicate, and homogenized in 200 µL of 0.85% saline solution. The solutions were added to Nutrient Agar (bacterial medium), Sabouraud Dextrose Agar (fungal medium), and Yeast Extract Peptone Dextrose (yeast medium). Media compositions are provided in Table S6. Isolated colonies were purified by single-colony streaking on the same medium and stored in 40% glycerol at −80°C.

### Taxonomic identification of microbial isolates

Microbial isolates were suspended in 40 µL of sterilized water and used for PCR amplification of the 16S rDNA (82), rpoB (83), and ITS (74). Primer sequences and PCR conditions are provided in Table S5. PCR products were sequenced by Macrogen Europe (France Genome Center, Roissy-en-France, France). Taxonomic identities of the isolates were determined using BLAST in Geneious Prime 2024.0.5 (https://www.geneious.com).

### PEITC tolerance of cultivable gut microorganisms

Microorganisms isolated and cultivated from the gut of *D. radicum* were grown in 11 mL of Nutrient Broth (NB) medium in Falcon tubes and incubated 20 h at 20°C under agitation (120 rpm). Cultures were then normalized to OD₆₀₀ = 0.01.

A 125 µM PEITC solution was prepared yielding a total volume of 100 µL. Subsequently, 100 µL of standardized cultures was added to the 96-well plate to obtain a final volume of 200 µL.

Two controls were included: (i) a growth control corresponding to 100 µL of culture (normalized to DO₆₀₀ = 0.01) and 100 µL of resazurin (2%) and (ii) a sterility control consisting of 200 µL of NB medium.

Bacterial growth over time was monitored using a millifluidics system (MilliDrop AzurEvo, https://www.goldstandarddiagnostics.com/instruments/millidrop-azurevo.html). In this system, microorganisms are incubated in 1 µL droplets, and growth is monitored by nephelometry (i.e., light scattering) every 30 min to estimate the cell density within each droplet. For each microbial isolate, three independent replicates were prepared with ten droplets each for a total of 30 growth curves per isolate. Growth curves were modeled from nephelometry measurements to evaluate tolerance to PEITC. Growth parameters were calculated using the “gcplyr” package (84) to compare the microbial responses between PEITC (125 µM) and control (0 µM) treatments. Three parameters were extracted: lag time (time before exponential growth begins), the midpoint (time to reach 50% of maximum growth), and the area under the curve (AUC, integral of the growth curve). Statistical comparisons between PEITC and control treatments were performed using Mann-Whitney tests for each microbial isolate.

### Detection of the *saxA* gene in microbial isolates

The extracted DNA used above for identification of microbial isolates was also used for the amplification of the *saxA* gene. Given the known sequence diversity among *saxA* homologs, four primer sets (CL1, CL2, CL3, and CL5) were used targeting different regions of the gene (85). Primer sequences and PCR conditions are provided in Table S5. PCR-positive products were sequenced by Macrogen Europe (France Genome Center, Roissy-en-France, France), translated into protein sequences using Geneious Prime 2024.0.5 (https://www.geneious.com) and compared to sequences with similar conserved domains using CD-search (86). *saxA* DNA sequences were compared to NCBI’s RefSeq database using BLAST, and the top 50 strains with *saxA* homologs were aligned with MAFFT. A maximum-likelihood phylogenetic tree was constructed using RAxML with 1,000 bootstrap replicates.

### ITC degradation by microbial isolates

To assess the ability of microbial isolates to degrade 2-phenylethyl isothiocyanate (PEITC), *saxA*-positive microbial isolates were cultured in M9 minimal media (see Table S6 for composition). Experimental assays were performed in five biological replicates in 50 mL of exponentially growing microbial cultures, each supplemented with 0.75 µL of PEITC (100 µM). Five negative control replicates (M9 medium with 0.75 µL of PEITC and without the microbial isolate), five positive control replicates (M9 medium with the microbial isolate), and one sterility control (M9 medium) were also included. Optic density at 600 nm for replicates with microbial isolate was measured, and samples of 500 µL were collected at intervals of 0 h (when PEITC was added to the medium), 3 h, 6 h, 12 h, and 24 h. Samples were centrifuged at 12,000 rpm for 5 min, and 400 µL of the supernatant was recovered. Then, 100 µL of liquid media was diluted in 100 µL of isopropanol (IPA) and 50 µL of n-acetyl-cysteine (0.1M) in KH_2_PO_4_ buffer (pH = 7). To ensure derivatization of PEITC, the samples were incubated for 2 h at 50°C under agitation (900 rpm). The samples were then filtered using a 0.22 µm PTFE membrane before analysis.

UHPLC-DAD analysis was performed on a Vanquish core system (Thermo Fisher Sci, Les Ulis, France). The separation of PEITC was done using a Hypersil Gold Aq 100 × 2.1 mm, 1.9 µm column (Thermo Fisher Sci) kept at 45°C.

Mobile phases consisted of A) water, 0.1% formic acid and B) ACN, 0.1% formic acid. Elution gradient was as follows: 30% B for 1 min, 50% B in 3 min, 95% B in 1 min, 95% B for 6 min, 30% B in 1 min and hold for 6 min. The total run time was 18 min. Injection volume was 2 µL. PEITC was identified based on retention time, UV-VIS spectra using a pure analytical standard as reference. Quantification was performed using absorbance at 278 nm and a five-point external calibration curve.

To test whether PEITC concentration declines more rapidly in the presence of a microbial isolate, values were transformed into proportions (0 and 1) and a generalized linear model was fitted using the glmmTMB function of the glmmTMB package (87). Type II Wald χ^2^ tests were conducted using the ANOVA function from the car package (69).

## Data Availability

All raw data sets are publicly available in the European Nucleotide Archive (ENA) of European Molecular Biology Laboratory’s European Bioinformatics Institute (EMBL-EBI) database system under project accession no PRJEB98871.

## SUPPLEMENTAL MATERIAL

Supplemental material msystems.01269-25-s0001.docx Tables S1 to S6 and Fig. S1 to S13.

ASM does not own the copyrights to Supplemental Material that may be linked to, or accessed through, an article. The authors have granted ASM a non-exclusive, world-wide license to publish the Supplemental Material files. Please contact the corresponding author directly for reuse.
