# Supplementary material for: Contrasting glucosinolate profiles in rapeseed genotypes shape the rhizosphere-insect continuum and microbial detoxification potential in a root herbivore
Source: mSystems. 2025 Nov 17;10(12):e01269-25. doi: 10.1128/msystems.01269-25 (PMC12710311; doi:10.1128/msystems.01269-25)
Supplement: Supplemental material — Tables S1 to S6 and Fig. S1 to S13. [file msystems.01269-25-s0001.docx]

**Figure S1:** Concentration of glucosinolates in roots expressed in micrograms per gram of dry weight (µg/g DW). The four modalities were derived from combining two rapeseed genotypes (GLS+ and GLS-) with three soil legacy conditions (GLS-, GLS+ and Wheat), resulting in the following four modalities: GLS-/Wheat, GLS-/GLS-, GLS+/Wheat and GLS+/GLS+. Data were analysed using the Kruskal-Wallis test. Different letters indicate a significant difference where *p* < 0.05.


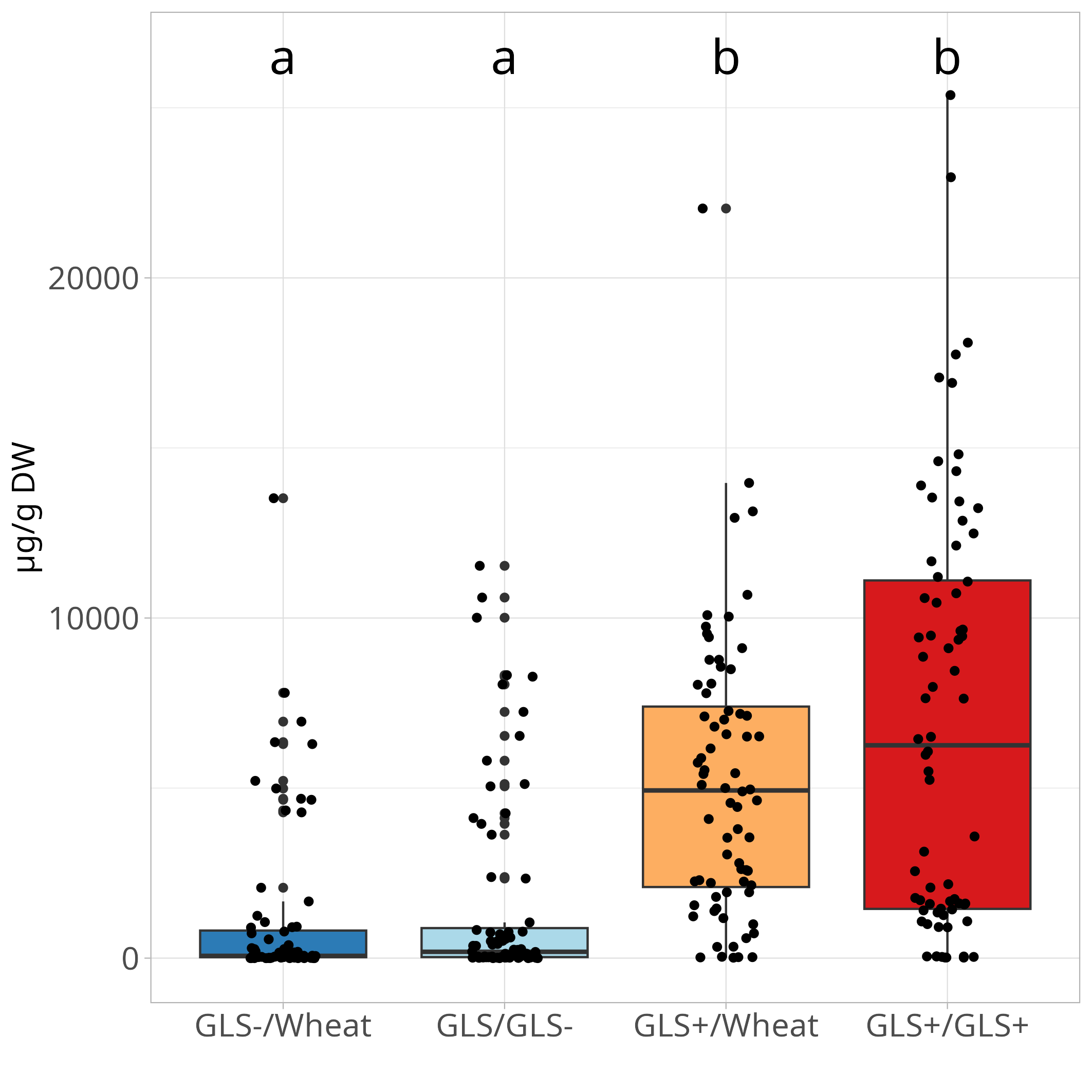

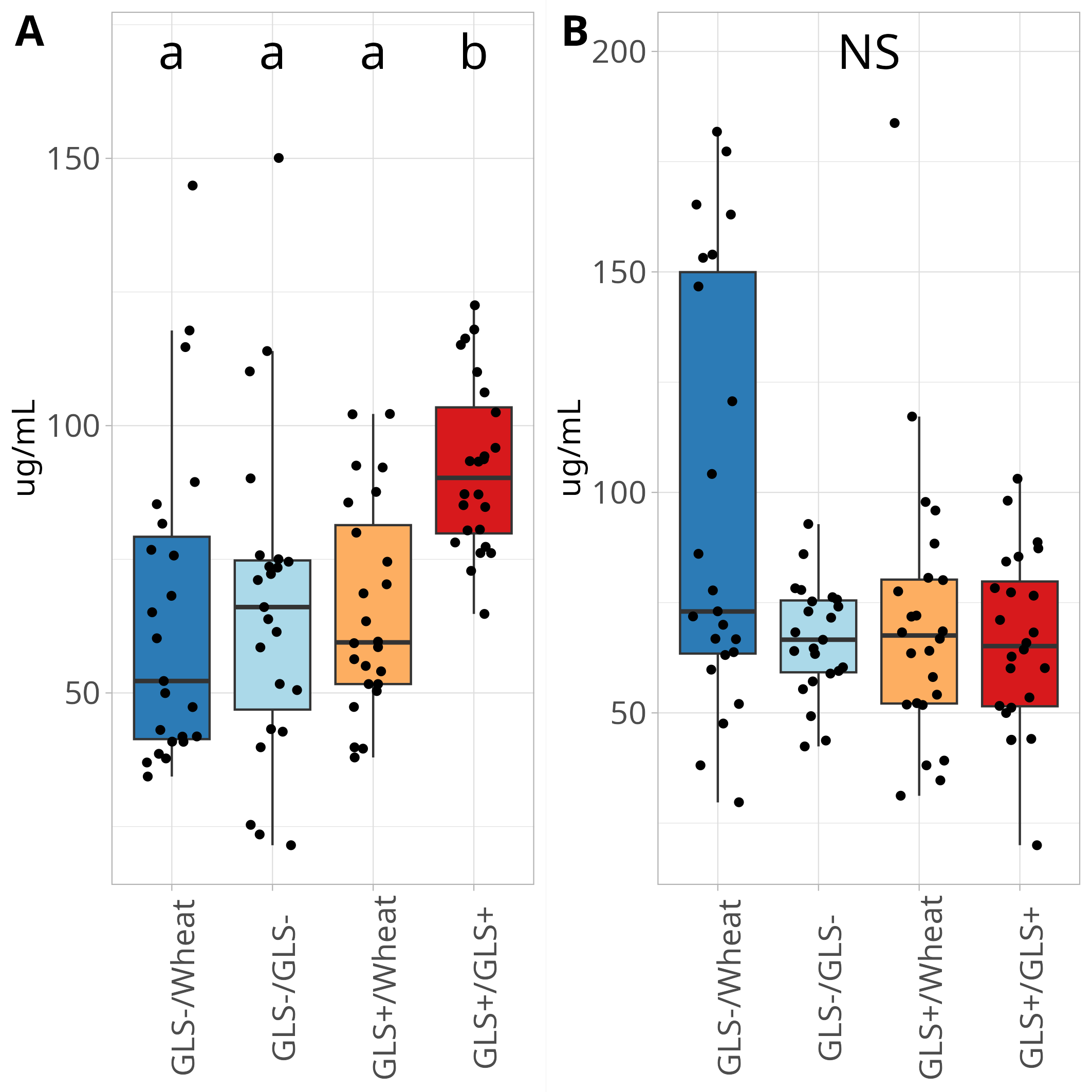


**Figure S2**: Concentration of digestible carbohydrates and soluble proteins in roots, expressed in µg/mL. The four modalities were derived from combining two rapeseed genotypes (GLS+ and GLS-) with three soil legacy conditions (GLS-, GLS+ and Wheat), resulting in the following four modalities: GLS-/Wheat, GLS-/GLS-, GLS+/Wheat and GLS+/GLS+. Data were analysed using the Kruskal-Wallis test. Different letters indicate a significant difference where *p* < 0.05.


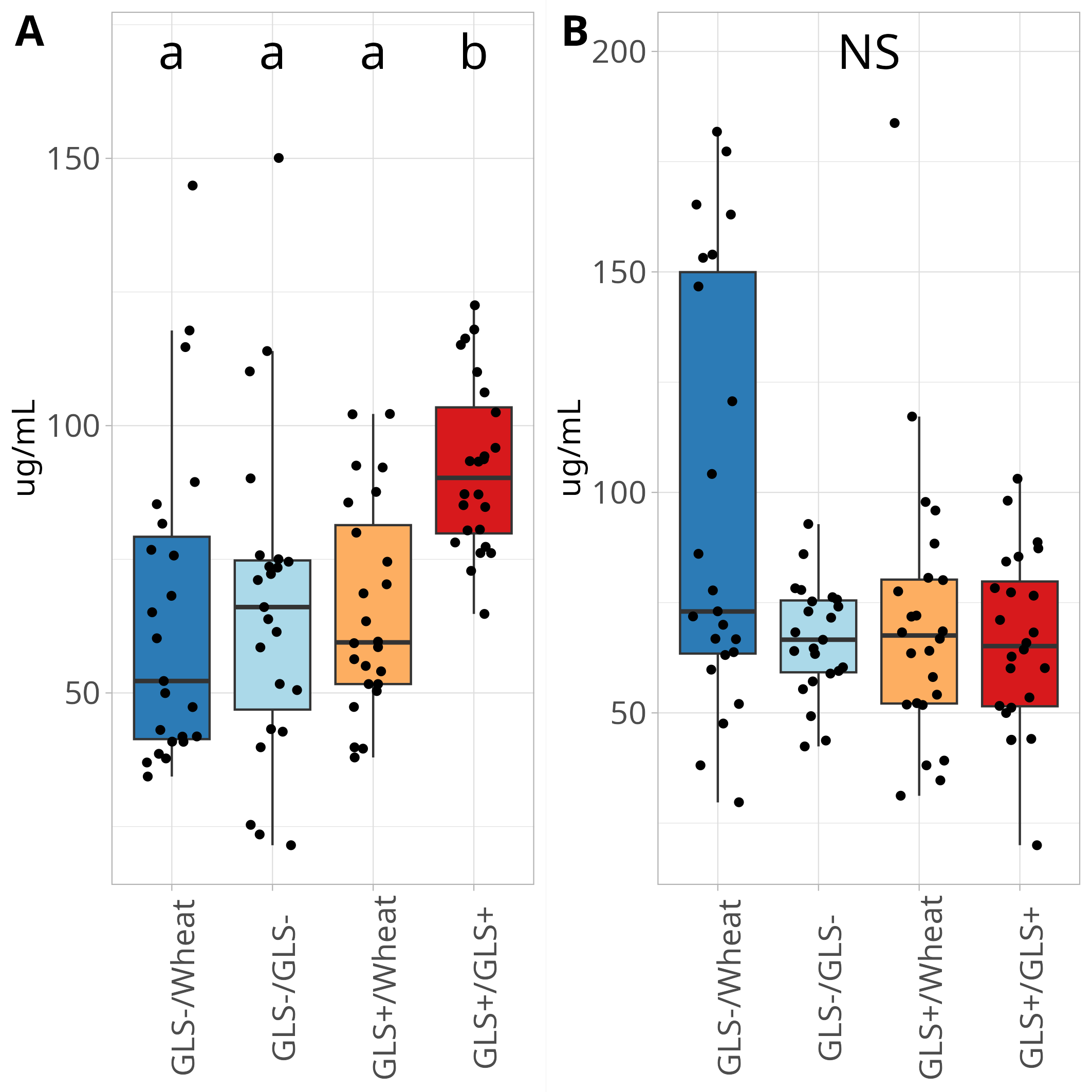


**Figure S3:** Relative abundance of bacterial communities of rhizospheric soil (A) and roots (B) at the phylum level. The four modalities were derived from combining two rapeseed genotypes (GLS+ and GLS-) with three soil legacy conditions (GLS-, GLS+ and Wheat), resulting in the following four modalities: GLS-/Wheat, GLS-/GLS-, GLS+/Wheat and GLS+/GLS+.


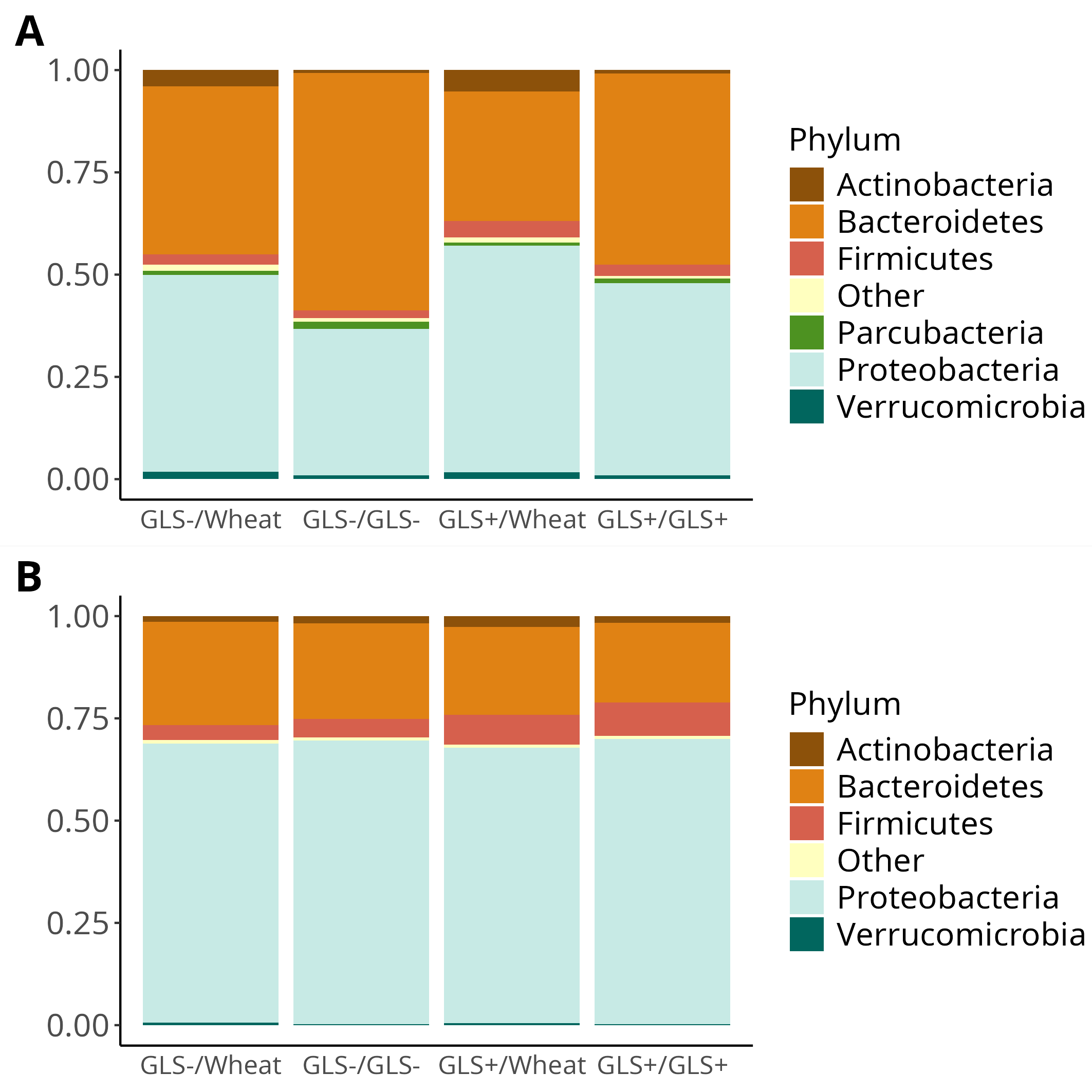


**Figure S4:** Relative abundance of fungal communities of rhizospheric soil (A) and roots (B) at the phylum level. The four modalities were derived from combining two rapeseed genotypes (GLS+ and GLS-) with three soil legacy conditions (GLS-, GLS+ and Wheat), resulting in the following four modalities: GLS-/Wheat, GLS-/GLS-, GLS+/Wheat and GLS+/GLS+.


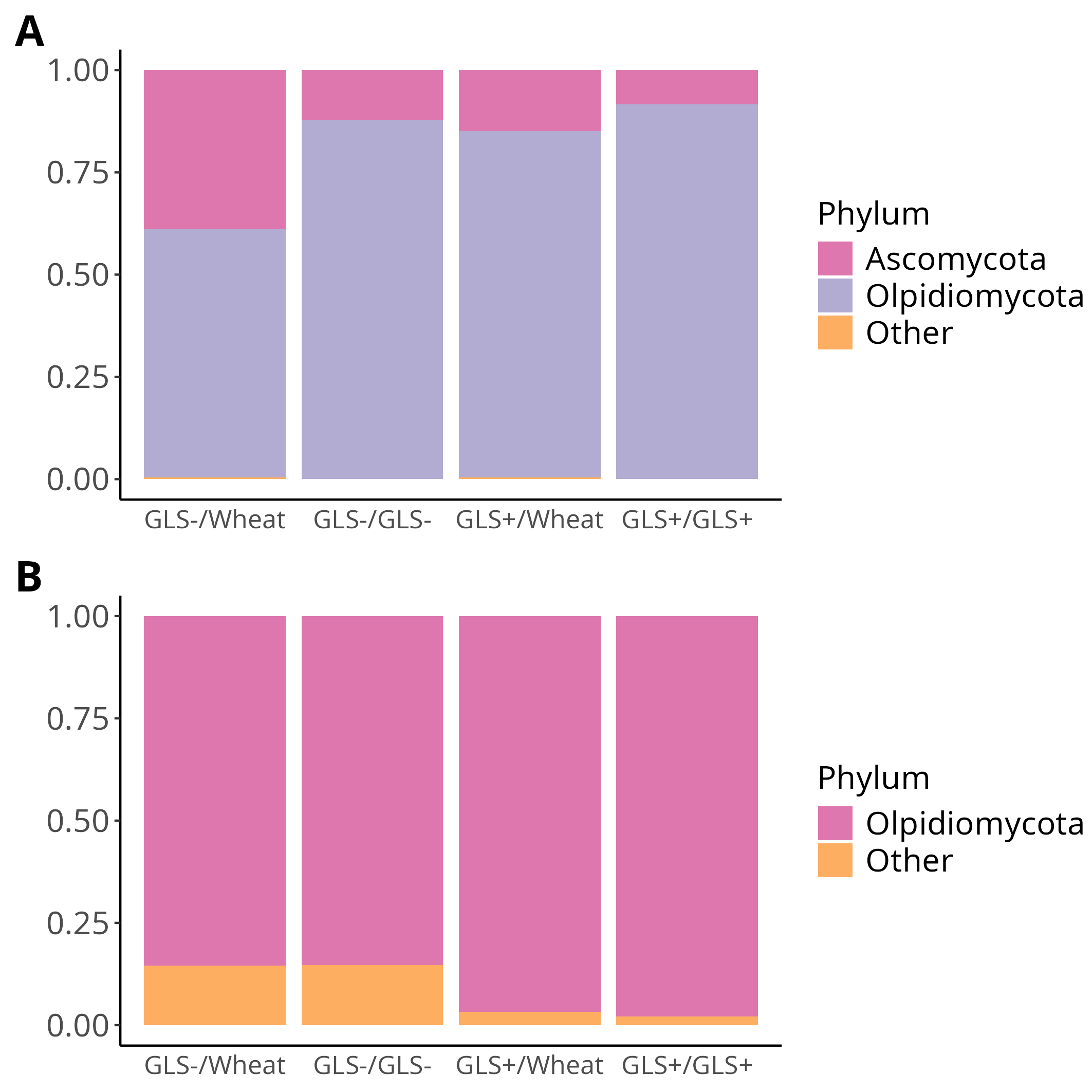


**Figure S5:** Observed ASVs and Shannon index of bacterial communities in larvae (A & C) and adults (B & D) of *Delia radicum* developping on *Brassica napus* growing in different soils. The four modalities tested correspond to the two rapeseed genotypes (GLS+ and GLS-) combined with soil legacy conditions (GLS-, GLS+ and Wheat) to obtain: GLS-/Wheat, GLS-/GLS-, GLS+/Wheat and GLS+/GLS+. Kruskal-Wallis and ANOVA test; Different letters indicate a significant difference, p<0.05


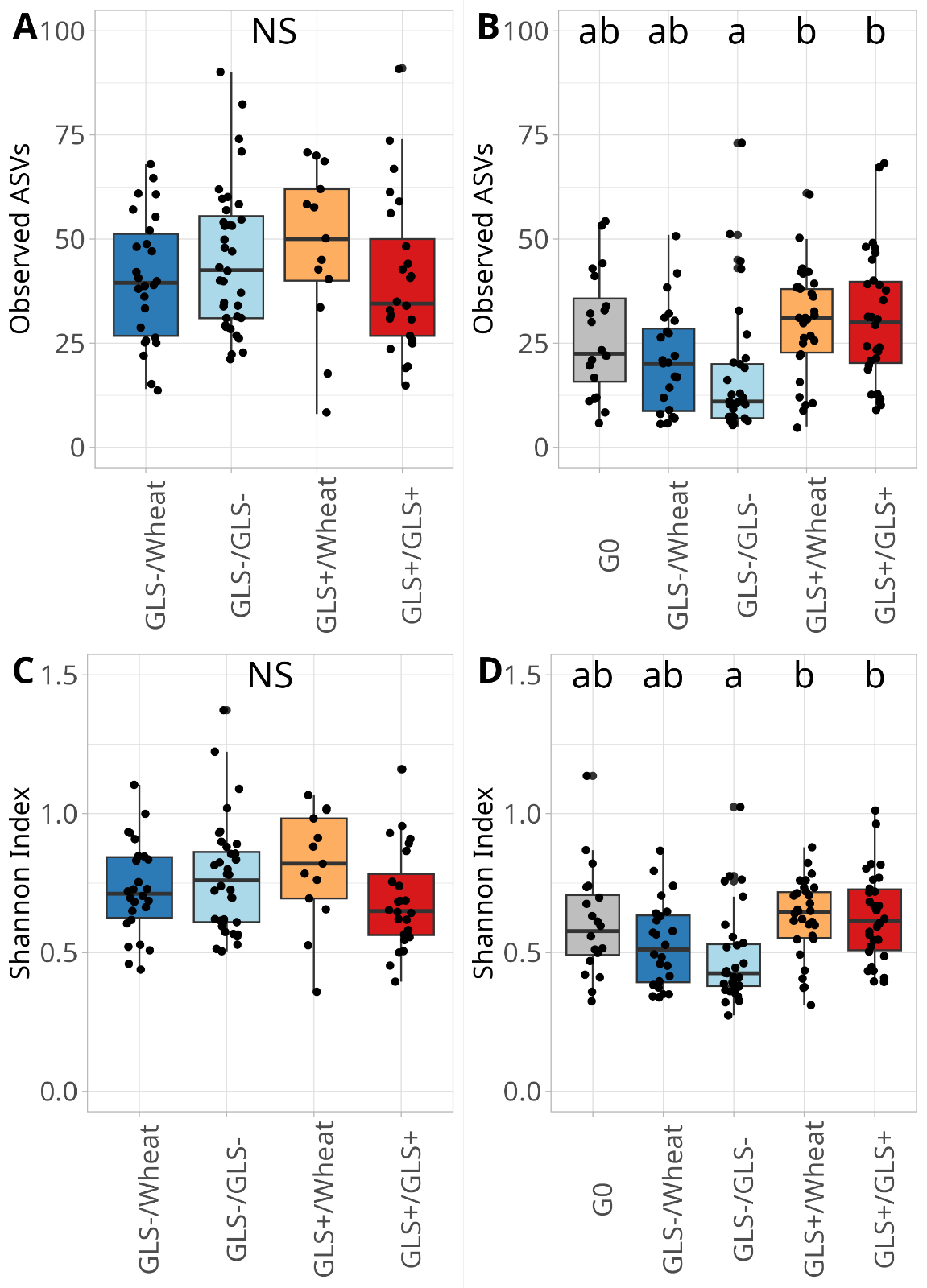


**Figure S6:** Observed ASVs and Shannon index of fungal communities in larvae (A & C) and adults (B & D) of *Delia radicum* developping on *Brassica napus* growing different soils. The four modalities tested correspond to the two rapeseed genotypes (GLS+ and GLS-) combined with soil legacy conditions (GLS-, GLS+ and Wheat) to obtain: GLS-/Wheat, GLS-/GLS-, GLS+/Wheat and GLS+/GLS+. Kruskal-Wallis and ANOVA test test; Different letters indicate a significant difference, p<0.05


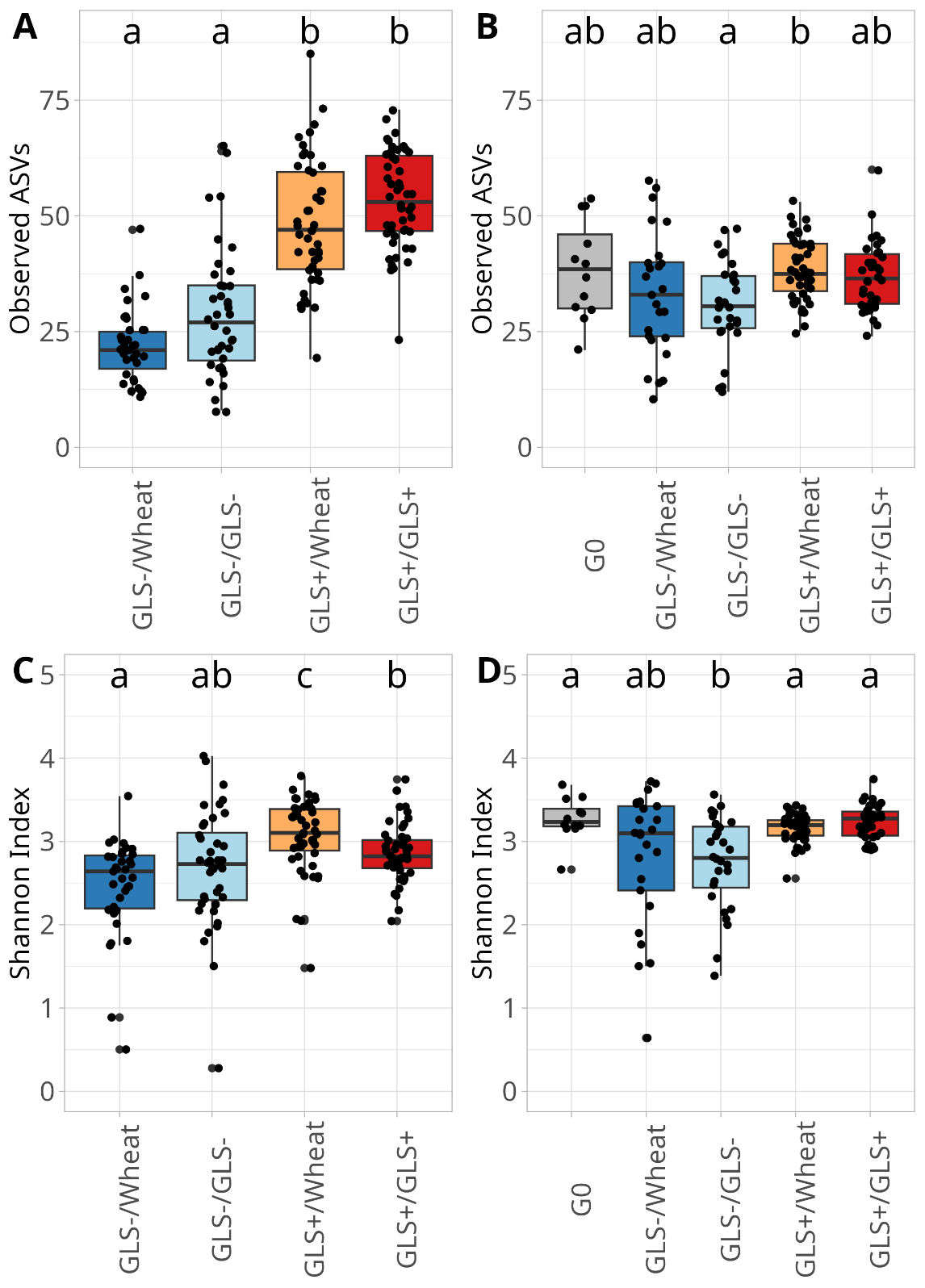


**Figure S7:** Relative abundance of bacterial communities of larvae (A) and adults (B) at the phylum level. The four modalities tested correspond to the two rapeseed genotypes (GLS+ and GLS-) combined with soil legacy conditions (GLS-, GLS+ and Wheat) to obtain: GLS-/Wheat, GLS-/GLS-, GLS+/Wheat and GLS+/GLS+.


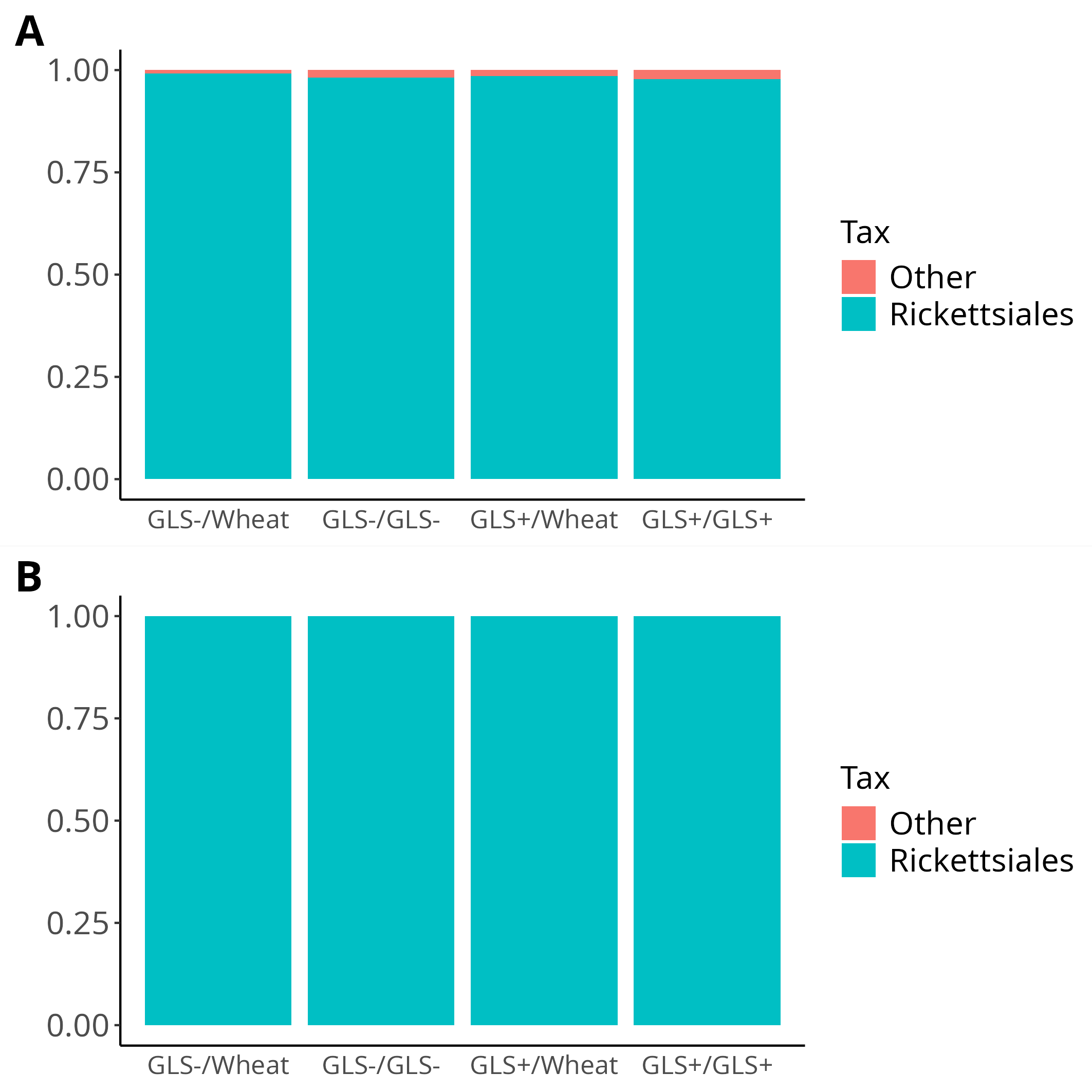


**Figure S8:** Relative abundance of fungal communities of larvae (A) and adults (B) at the phylum level. The four modalities tested correspond to the two rapeseed genotypes (GLS+ and GLS-) combined with soil legacy conditions (GLS-, GLS+ and Wheat) to obtain: GLS-/Wheat, GLS-/GLS-, GLS+/Wheat and GLS+/GLS+.


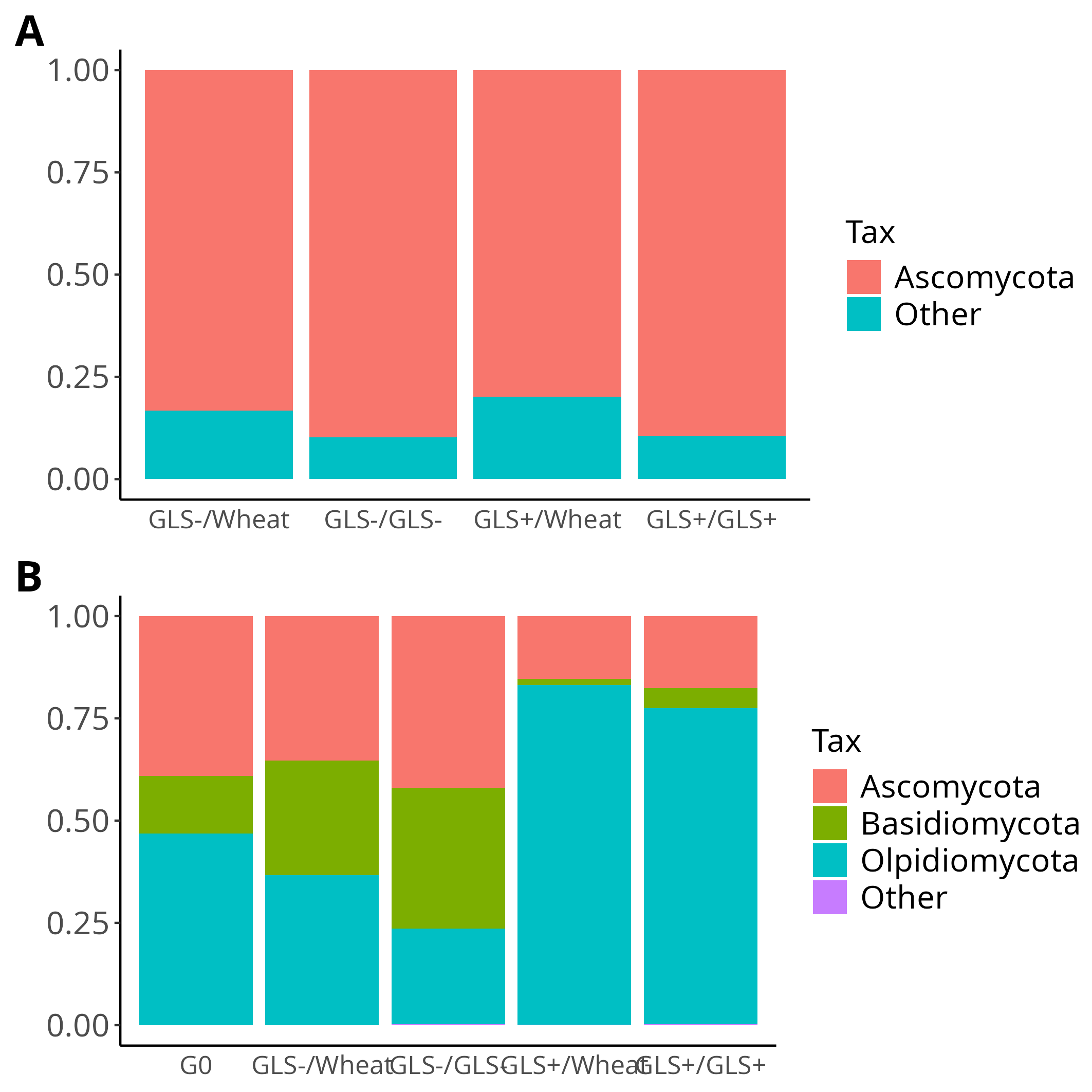


**Table S1:** List of shared bacteria species (soil, root and larvae) for each modality. The four modalities tested correspond to the two rapeseed genotypes (GLS+ and GLS-) combined with soil legacy conditions (GLS-, GLS+ and Wheat) to obtain: GLS-/Wheat, GLS-/GLS-, GLS+/Wheat and GLS+/GLS+.

| **Combination** | **Species** |
| --- | --- |
| GLS+/GLS+ | *Pantoea agglomerans* |
| GLS+/GLS+ | *Bacillus circulans* |
| GLS+/GLS+ | *Pseudomonas kilonensis* |
| GLS+/Wheat | *Pseudomonas fluorescens* |
| GLS+/Wheat | *Paenibacillus polymyxa* |
| GLS+/Wheat | *Lelliottia* sp. |
| GLS+/Wheat | *Stenotrophomonas* sp. |
| GLS-/GLS- | *Acinetobacter johnsonii* |
| GLS-/GLS- | *Agrobacterium fabrum* |
| GLS-/GLS- | *Clostridium arbusti* |
| GLS-/GLS- | *Paenibacillus odorifer* |
| GLS-/GLS- | *Chryseobacterium* sp. |
| GLS-/GLS- | *Stenotrophomonas maltophilia* |
| GLS-/GLS- | *Devosia* sp. |
| GLS-/GLS- | *Aeromonas encheleia* |
| GLS-/GLS- | *Chryseobacterium luteum* |
| GLS-/GLS- | *Bacillus* sp. |
| GLS-/GLS- | *Rhodoferax* sp. |
| GLS-/Wheat | *Sphingobacterium* sp. |
| GLS-/Wheat | *Pseudoxanthomonas* sp. |
| GLS-/Wheat | *Stenotrophomonas rhizophila* |
| GLS-/Wheat | *Erwinia iniecta* |
| GLS+/GLS+ & GLS-/GLS- | *Delftia* sp. |
| GLS+/GLS+ & GLS-/GLS- | *Pseudomonas putida* |
| GLS+/GLS+ & GLS-/GLS- | *Duganella* sp. |
| GLS+/GLS+ & GLS-/Wheat | *Legionella* sp. |
| GLS+/Wheat & GLS-/GLS- | *Paenibacillus amylolyticus* |
| GLS+/Wheat & GLS-/GLS- | *Flavobacterium* sp. |
| GLS+/Wheat & GLS-/GLS- | *Leclercia* sp. |
| GLS+/Wheat & GLS-/Wheat | *Serratia plymuthica* |
| GLS-/GLS- & GLS-/Wheat | *Paenibacillus borealis* |
| GLS+/GLS+ & GLS+/Wheat & GLS-/GLS- | *Achromobacter* sp. |
| GLS+/GLS+ & GLS-/GLS- & GLS-/Wheat | *Comamonas* sp. |
| GLS+/Wheat & GLS-/GLS- & GLS-/Wheat | *Agrobacterium tumefaciens* |
| GLS+/GLS+ & GLS+/Wheat & GLS-/GLS- & GLS-/Wheat | *Arthrobacter* sp. |
| GLS+/GLS+ & GLS+/Wheat & GLS-/GLS- & GLS-/Wheat | *Pseudomonas frederiksbergensis* |
| GLS+/GLS+ & GLS+/Wheat & GLS-/GLS- & GLS-/Wheat | *Pseudomonas* sp. |
| GLS+/GLS+ & GLS+/Wheat & GLS-/GLS- & GLS-/Wheat | *Pseudomonas brassicacearum* |
| GLS+/GLS+ & GLS+/Wheat & GLS-/GLS- & GLS-/Wheat | *Microbacterium* sp. |
| GLS+/GLS+ & GLS+/Wheat & GLS-/GLS- & GLS-/Wheat | *Acinetobacter calcoaceticus* |
| GLS+/GLS+ & GLS+/Wheat & GLS-/GLS- & GLS-/Wheat | *Clostridium* sp. |
| GLS+/GLS+ & GLS+/Wheat & GLS-/GLS- & GLS-/Wheat | *Paenibacillus* sp. |

**Table S2:** List of shared fungal species (soil, root and larvae) for each modality. The four modalities tested correspond to the two rapeseed genotypes (GLS+ and GLS-) combined with soil legacy conditions (GLS-, GLS+ and Wheat) to obtain: GLS-/Wheat, GLS-/GLS-, GLS+/Wheat and GLS+/GLS+.

| **Combination** | **Species** |
| --- | --- |
| GLS+/GLS+ | *Candida vartiovaarae* |
| GLS+/Wheat | *Arthrobotys oudemansii* |
| GLS+/GLS+ & GLS+/Wheat | *Fusarium waltergamsii* |
| GLS+/GLS+ & GLS+/Wheat | *Thelonectria* sp. |
| GLS+/GLS+ & GLS+/Wheat | *Fusicolla septimanifiniscientiae* |
| GLS+/GLS+ & GLS+/Wheat | *Ilyonectria macrodidyma* |
| GLS+/GLS+ & GLS+/Wheat & GLS-/GLS- & GLS-/Wheat | *Fusarium acutatum* |
| GLS+/GLS+ & GLS+/Wheat & GLS-/GLS- & GLS-/Wheat | *Olpidium brasscae* |

**Figure S9:** Variation in lag time of microorganisms at 125 µM of PEITC compared with the mean of the PEITC-free control. The red line corresponds to the control mean for each microorganism species (ratio = 1). Values above 1 indicate a higher lag time than the control mean, while values below 1 indicate a reduced lag time. Mann-Whitney tests were performed in pairs between the control (PEITC concentration = 0 µM) and the condition with 125 µM of PEITC (ns = not significant, * = p<0.05, ** = p<0.01, *** = p<0.001).


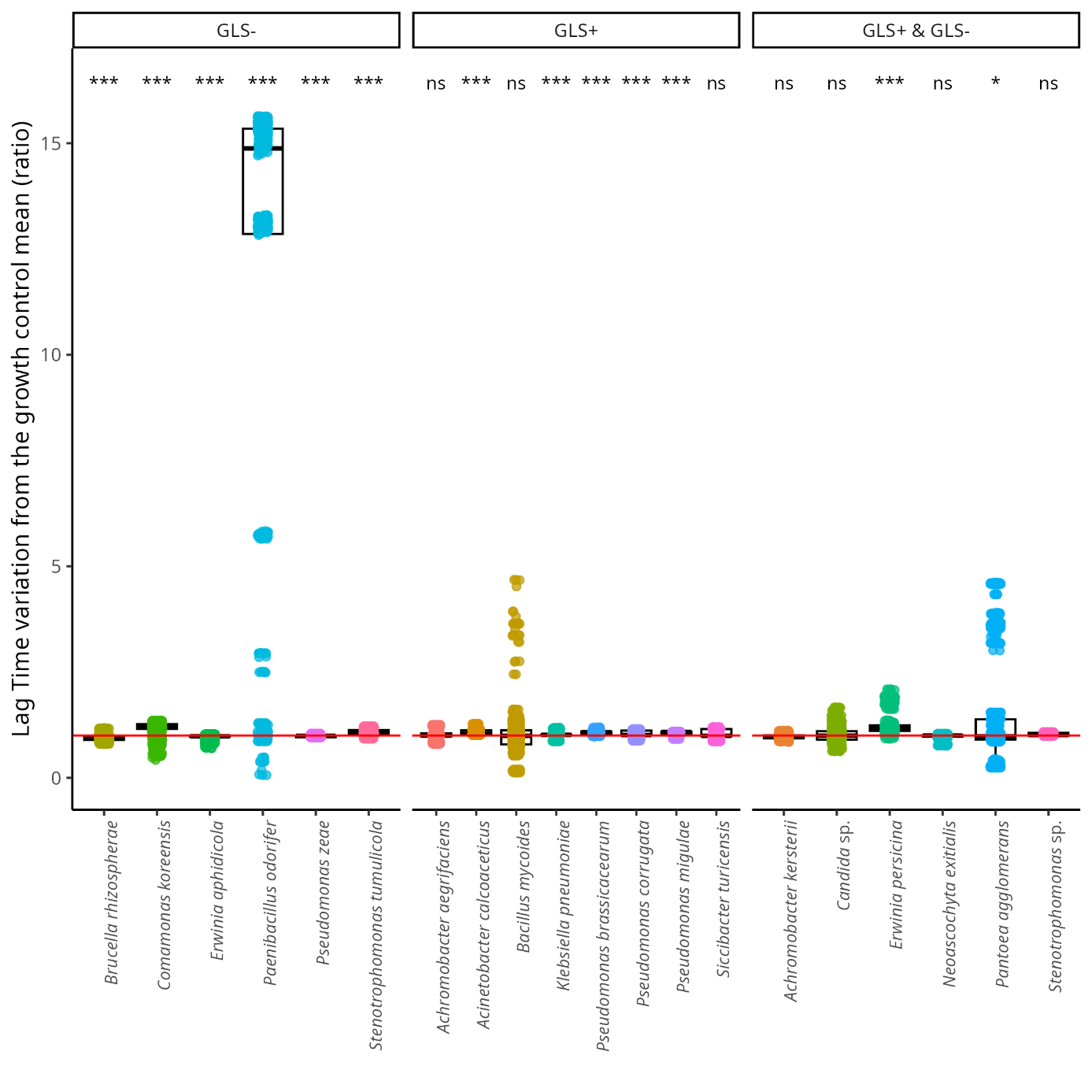


**Figure S10:** Variation in midpoint of microorganisms at 125 µM of PEITC compared with the mean of the PEITC-free control. The red line corresponds to the control mean for each microorganism species (ratio = 1). Values above 1 indicate a higher midpoint than the control mean, while values below 1 indicate a reduced midpoint. Mann-Whitney tests were performed in pairs between the control (PEITC concentration = 0 µM) and the condition with 125 µM of PEITC (ns = not significant, * = p<0.05, ** = p<0.01, *** = p<0.001).


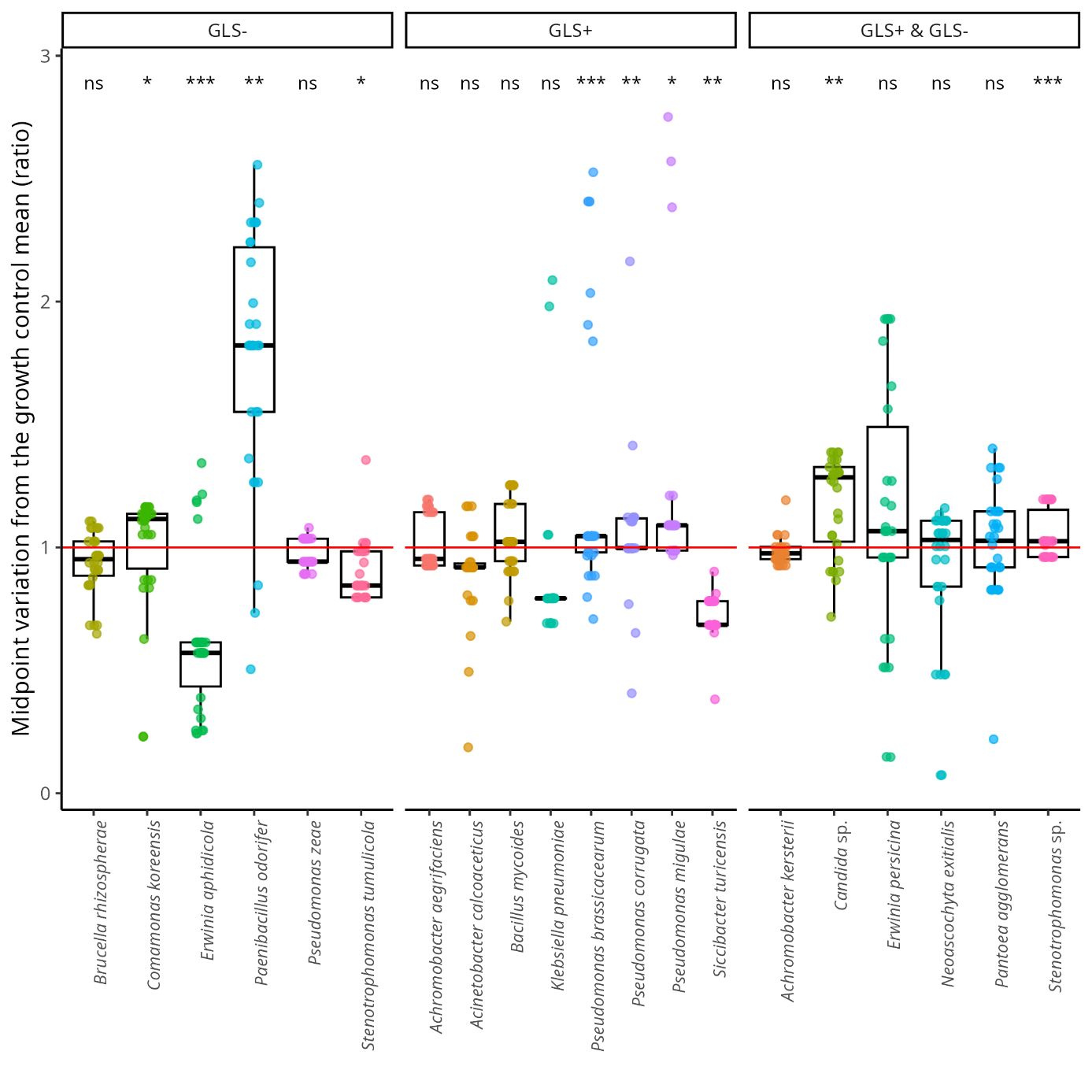


**Figure S11:** Area under the curve (AUC) calculated from the bacterial growth extracted from larvae guts that have grown on GLS+ or GLS- rapeseed as a function of PEITC concentration (n = 30 per species). Mann-Whitney tests were performed in pairs between the control (PEITC concentration = 0 µM) and the condition with 125 µM of PEITC (ns = not significant, * = p<0.05, ** = p<0.01, *** = p<0.001). Black vertical bars represent standard deviation.


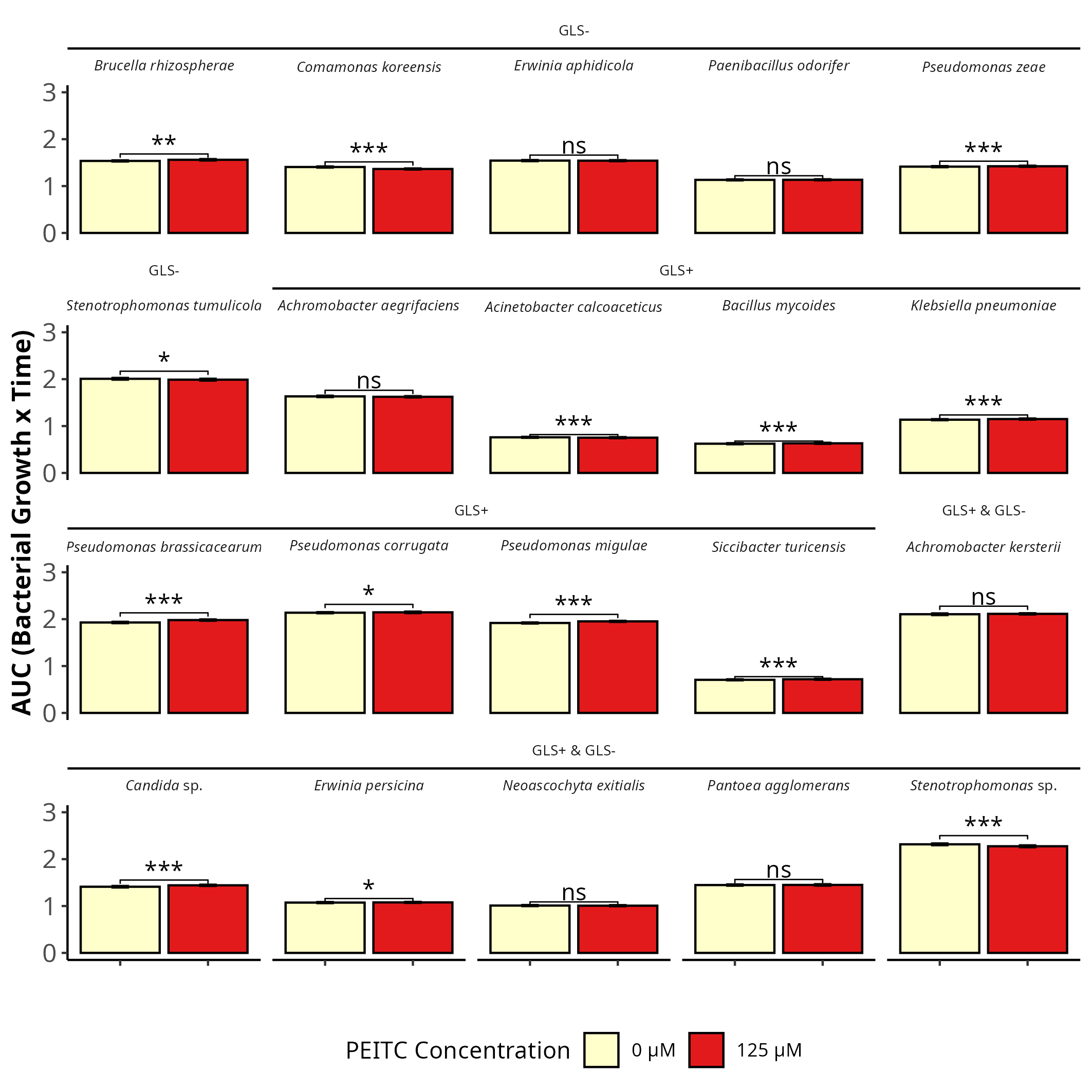


**Figure S12:** Lag Time calculated from the bacterial growth extracted from larvae guts that have grown on GLS+ or GLS- rapeseed as a function of PEITC concentration (n = 30 per species). Mann-Whitney tests were performed in pairs between the control (PEITC concentration = 0 µM) and the condition with 125 µM of PEITC (ns = not significant, * = p<0.05, *** = p<0.001). Black vertical bars represent standard deviation.


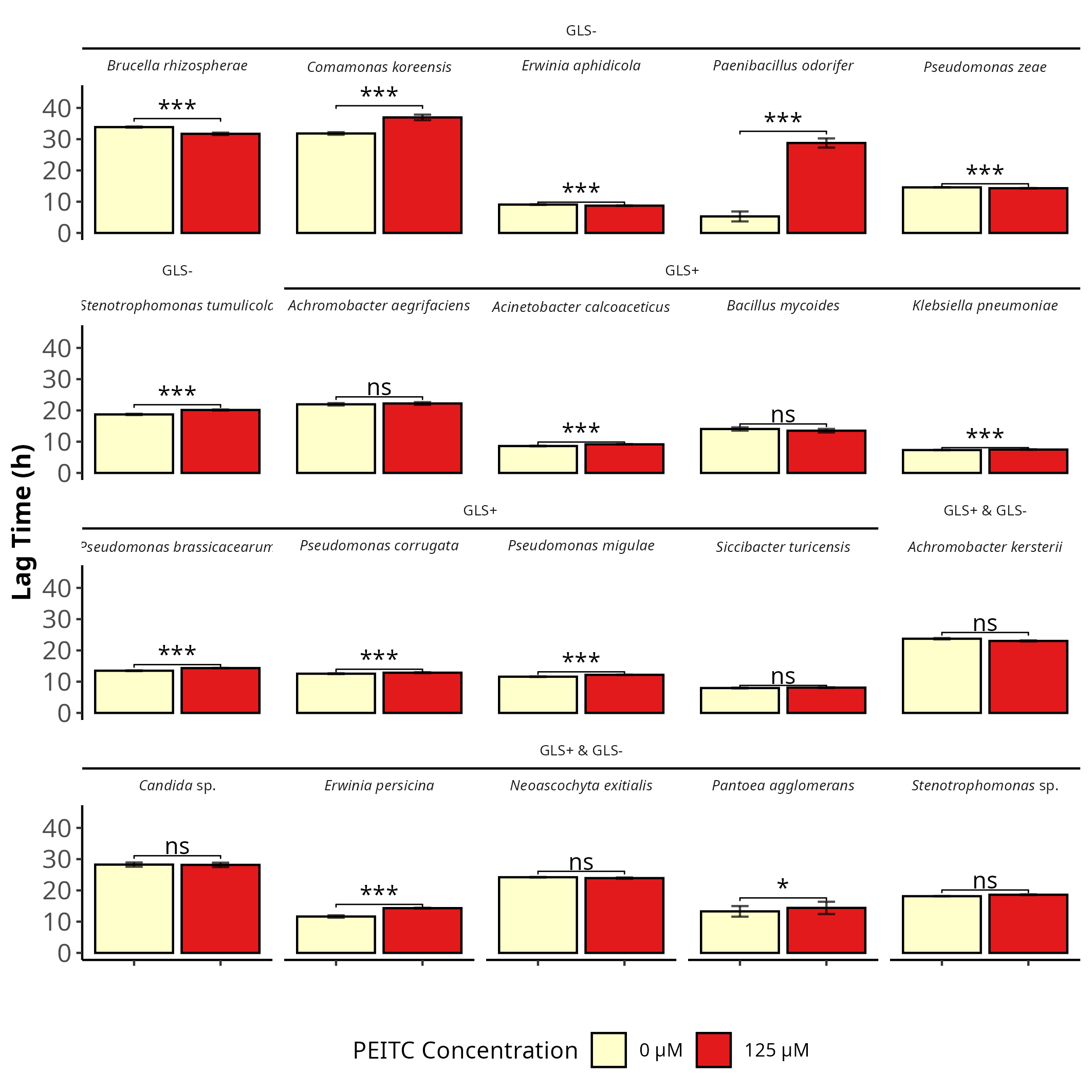


**Figure S13:** Midpoint calculated from the bacterial growth extracted from larvae guts that have grown on GLS+ or GLS- rapeseed as a function of PEITC concentration (n = 30 per species). Mann-Whitney tests were performed in pairs between the control (PEITC concentration = 0 µM) and the condition with 125 µM of PEITC (ns = not significant, * = p<0.05, ** = p<0.01, *** = p<0.001). Black vertical bars represent standard deviation.


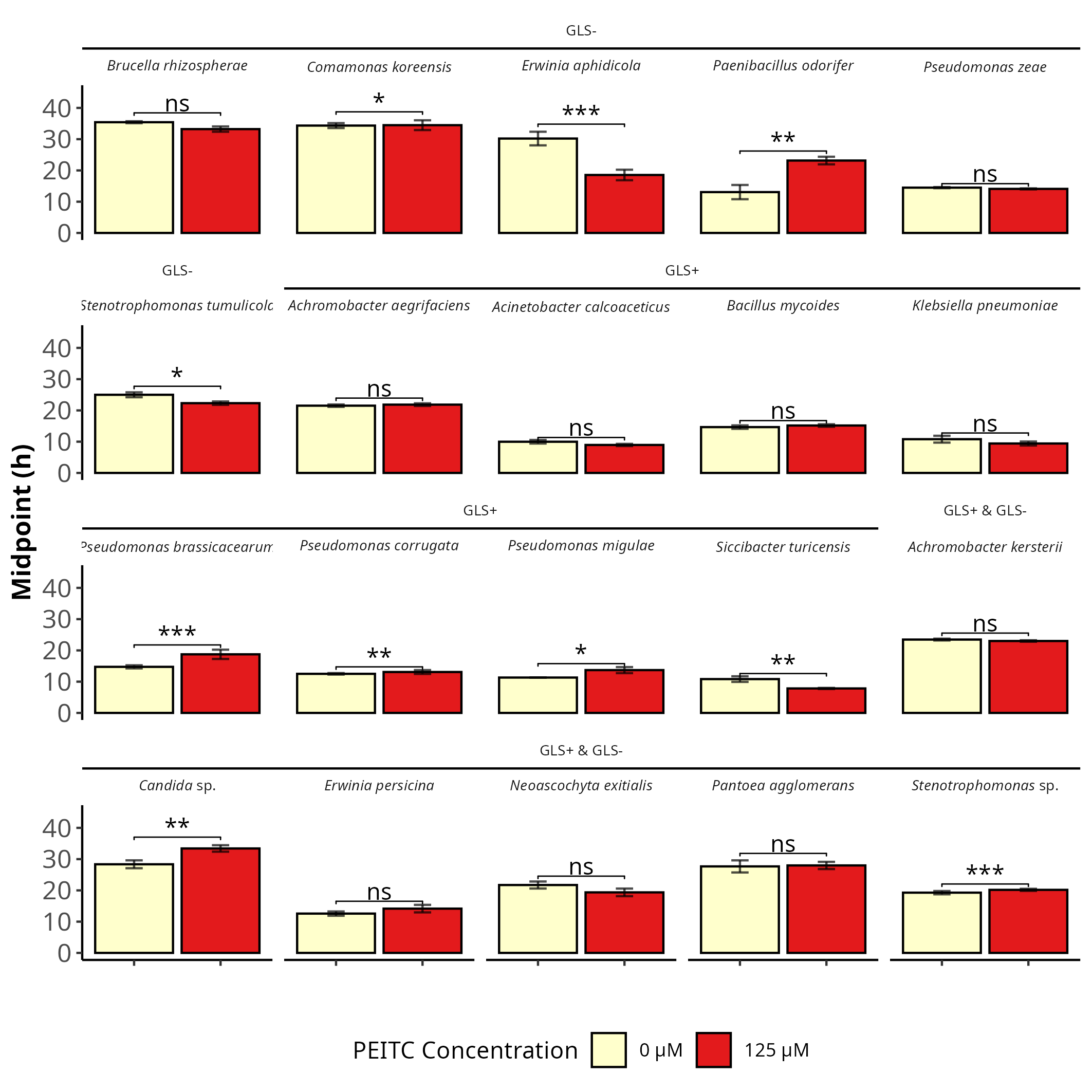


**Table S3:** Elution gradient used for GLSs separation (Solvent A: Water, 0.1 % Formic Acid; solvent B: ACN, 0.1% Formic Acid).

| Minutes | Flow (µl/min) | % Solvent A | % Solvent B |
| --- | --- | --- | --- |
| 0 | 0.4 | 98 | 2 |
| 3 | 0.4 | 91 | 9 |
| 12 | 0.4 | 83 | 17 |
| 13 | 0.4 | 80 | 20 |
| 24 | 0.4 | 68 | 32 |
| 26 | 0.4 | 10 | 90 |
| 29 | 0.4 | 10 | 90 |
| 30 | 0.4 | 98 | 2 |
| 31 | 0.4 | 98 | 2 |

**Table S4**: Glucosinolates used for quantification

| m/z trace (negative mode) | Name | Raw formula |
| --- | --- | --- |
| 422 | Glucoiberin | *C11H21NO10S3* |
| 420 | Glucoerucin | *C12H23NO9S3* |
| 447 | Glucobrassicin | *C16H20N2O9S2* |
| 422 | Gluconasturtiin | *C15H21NO9S2* |
| 434 | Glucoraphenin | *C12H21NO10S3* |
| 436 | Glucoraphanin | *C12H23NO10S3* |
| 386 | Glucobrassicanapin | *C12H21NO9S2* |
| 358 | Sinigrin H2O | *C10H16NO9S2* |
| 450 | Glucoalyssin | *C13H25NO10S3* |
| 372 | Gluconapin | *C11H18NO9S2* |
| 388 | Progoitrin | *C11H19NO10S2* |

**Table S5:** Primers and PCR conditions for amplicon library construction (gyrB, ITS), molecular identification of microbial strains (rpoB, ITS, 16S) extracted from *D. radicum* gut and PCR identification of *saxA* clusters (CL1-saxA, CL2-saxA, CL3-saxA, CL5-saxA)

| Primer | Forward | Reverse | PCR conditions | | |
| --- | --- | --- | --- | --- | --- |
| gyrB | 5' - MGNCCNGSNATGTAYATHGG - 3' | 5' - ACNCCRTGNARDCCDCCNGA - 3' | 94°C | 3 min |  |
|  |  |  | 94°C | 30 sec | 35 cycles |
|  |  |  | 55°C | 45 sec |  |
|  |  |  | 70°C | 1 min 30 |  |
|  |  |  | 70°C | 10 min |  |
| ITS | 5' - GGTCATTTAGAGGAAGTAA - 3' | 5' - **G**TTYRCKRCGTTCTTCATCG - 3' | 94°C | 3 min |  |
|  |  |  | 94°C | 30 sec | 35 cycles |
|  |  |  | 50 °C | 45 sec |  |
|  |  |  | 72 °C | 1 min 30 |  |
|  |  |  | 72 °C | 10 min |  |
| 16S | 5' - GCCTACGGGNGGCWGCAG - 3' | 5' - GGACTACHVGGGTATCTAATCC - 3' | 95°C | 5 min |  |
|  |  |  | 95°C | 40 sec | 25 cycles |
|  |  |  | 55°C | 2 min |  |
|  |  |  | 72°C | 1 min |  |
|  |  |  | 72°C | 7min |  |
| rpoB | 5' - GGYTWYGAAGTNCGHGACGTDCA - 3' | 5' - TGACGYTGCATGTTBGMRCCCATMA - 3' | 95°C | 5 min |  |
|  |  |  | 95°C | 30 sec | 40 cycles |
|  |  |  | 58°C | 1 min |  |
|  |  |  | 72°C | 1 min 30 |  |
|  |  |  | 72°C | 5 min |  |
| CL1-saxA | 5' - ACGCAGGATGAAAAAGA - 3' | 5' - GGGTAAGGAGGCAATGA - 3' | 95°C | 5 min |  |
|  |  |  | 95°C | 30 sec | 30 cycles |
|  |  |  | 55°C | 1 min |  |
|  |  |  | 72°C | 30 sec |  |
|  |  |  | 75°C | 5 min |  |
| CL2-saxA | 5' - TGCAGCAAGTTCGTAACG - 3' | 5' - ACATCCTCYTCMCCCAT - 3' | 95°C | 5 min |  |
|  |  |  | 95°C | 30 sec | 30 cycles |
|  |  |  | 55°C | 1 min |  |
|  |  |  | 72°C | 30 sec |  |
|  |  |  | 75°C | 5 min |  |
| CL3-saxA | 5' - ATCTGCGTAAYCCKCTGGT - 3' | 5' - TTTAACACCACCACATCAGG - 3' | 95°C | 5 min |  |
|  |  |  | 95°C | 30 sec | 30 cycles |
|  |  |  | 55°C | 1 min |  |
|  |  |  | 72°C | 30 sec |  |
|  |  |  | 75°C | 5 min |  |
| CL5-saxA | 5' - ACTGGAATTTGGTGGAAAA - 3' | 5' - TAGTCACGSAGTTCAGCA - 3' | 95°C | 5 min |  |
|  |  |  | 95°C | 30 sec | 30 cycles |
|  |  |  | 55°C | 1 min |  |
|  |  |  | 72°C | 30 sec |  |
|  |  |  | 75°C | 5 min |  |

**Table S6:** Medium composition for isolation of larvae gut microorganisms

|  | Bacteria - NB | Bacteria - SDA | Yeast and Fungi - YEPD | M9 | M9 salts (5X) |
| --- | --- | --- | --- | --- | --- |
| Medium component | 0.3% meat extract | 1% bacto peptone | 1% yeast extract | 20% M9 salts (5X) | 4,3% Na_2_HPO_4_ ·2H_2_O |
|  | 0.5% meat peptide | 4% dextrose | 2% bacto peptone | 2% Glucose (20%) | 1,5% KH_2_PO_4_ |
|  | 2% agar | 1.5% bacto agar | 2% glucose | 0,2% MgSO_4_ (1M) | 0,23% NaCl |
|  |  |  | 2% agar | 0,01% CaCl_2_ (1M) | 0,5 % NH_4_Cl |
| pH | pH 6.8 | pH 5.6 | pH 6.5 | pH 7 |  |
| Antibiotic and fungicide | 100 mg.L^-1^ of cycloheximide | 100 mg.L^-1^ of ampiciline | 100 mg.L^-1^ of chloramphenicol |  |  |
|  |  | 5 mg.L^-1^ of gentamicine |  |  |  |
|  |  | 100 mg.L^-1^ of chloramphenicol |  |  |  |
